# Highly Water‐Stable 2D MOF as Dual Sensor for the Ultra‐Sensitive Aqueous Phase Detection of Nitrofuran Antibiotics and Organochlorine Pesticides

**DOI:** 10.1002/smll.202409095

**Published:** 2024-11-20

**Authors:** Supriya Mondal, Rupam Sahoo, Madhab C. Das

**Affiliations:** ^1^ Department of Chemistry Indian Institute of Technology Kharagpur Kharagpur West Bengal 721302 India

**Keywords:** aqueous medium sensing, multi‐responsive MOF, nitrofuran antibiotics, organochlorine pesticides, water‐stable MOF

## Abstract

Misuse of antibiotics and pesticides has led to hazardous effects on human health, livestock, agriculture, and aquaculture, which urges researchers to find simple, rapid, efficient, and cost‐effective methods for quick on‐site analysis of these organic pollutants with functional materials. Herein, a 2D chemically robust MOF: **IITKGP‐71**, {[Cd(MBPz)(2,6‐NDC)]·2H_2_O}*_n_* is strategically developed with ease in scalability and exploited as dual sensors toward the toxic antibiotic and pesticide detection via luminescence quenching in *aqueous medium*. The framework displays exceptional chemical robustness in water for 3 months, in an open atmosphere over 2 months, and wide range of aqueous pH solution (pH = 3–12) for a day. **IITKGP‐71** can selectively quench the nitrofuran antibiotics (NFZ and NFT) and organochlorine pesticide DCN while remaining unaffected by other interfering antibiotics and pesticides, respectively. An excellent *trade‐off* between high effectivity (high K_sv_) and high sensitivity (low LOD) was achieved for the targeted analytes. The easy scalability, high chemical stability, fast responsivity, multi‐responsive nature, recyclability with outstanding structural stability made this framework viable in playing a crucial role in safeguarding aquatic ecosystems and public health from the hazardous effects of antibiotics and pesticides.

## Graphical abstract

A chemically robust (aqueous pH = 3‐12 for a day and 3 months in water) Cd‐MOF (**IITKGP‐71**) is strategically developed for ultrasensitive detection of Nitrofuran (NFZ and NFT) antibiotics and DCN pesticide with high selectivity, rapid responsiveness and excellent recyclability in *aqueous medium*.

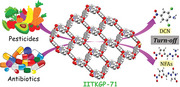

## 1 Introduction

Population growth, improvement of quality of life, and advancement in the modern health care systems have resulted in an increased level of various pharmaceuticals particularly antibiotics and toxic pesticides released into the environment. Nitrofuran antibiotics (NFAs) are commonly used in medical treatment for human and veterinary infection cures.^[^ ^1^, ^2^ ^]^ As humans and animals can only absorb 30% of antibiotics, the inefficient metabolism in living organisms leads to the excretion of the ingested residues in water and soil environments.^[^ ^3^, ^4^ ^]^ Additionally, large amounts of antibiotics are detected in wastewater generated by the pharmaceutical industry and sewage treatment plants, which led to a growing number of multi‐resistance bacteria in aquatic habitats. Thus, this large‐scale production and consumption of antibiotics along with their persistence in aquatic environments for a long time has not only brought potential risk to human health but also has detrimental effects on the ecosystem.^[^ ^5^ ^]^ Moreover, the spreading of antibiotic‐resistant genes can reduce the effectiveness of bacterial infection treatment.^[^ ^6^, ^7^ ^]^ Therefore, efficient detection of this specific organic pollutant residue from contaminated water bodies and wastewater streams has become a hot topic of global concern recently.

On the other hand, organochlorine pesticides (OCPs) are widely used in agriculture for the mass production of cereals, fruits, and vegetables in a short time.^[^ ^8^, ^9^, ^10^ ^]^ However, their irrational use led to bioaccumulation of the waste residues (≈99%) in the environment, which causes serious damage to ecological security, and ultimately jeopardizes the quality of human life and health through the food chain. 2,6‐dichloro‐4‐nitroaniline (DCN) or dichloran is one such broad‐spectrum OCP belonging to toxicity class IV.^[^ ^11^, ^12^, ^13^ ^]^ It has been extensively employed as an agricultural fungicide to treat wheat powdery mildew, and cotton rotten bell diseases, and prevent fruit trees and vegetables from being rotten. Nevertheless, DCN degrades slowly and can be sustained for a long time under environmental conditions, thus increasing the risk of human cell mutation and carcinogenicity. In addition, it can enter the human body through the skin, lungs, and the absorption from the gut wall, leading to convulsions and hyperreflexia, which makes it imperative to explore smart probes for selective and sensitive sensing of this pesticide.

Before the emergence of luminescence, the major techniques that have been explored for the detection of antibiotics (including nitrofuran class) and pesticides (including organochlorines) from aqueous environments include high‐performance liquid chromatography‐mass spectrometry, electrochemical methods, liquid chromatography‐tandem mass spectrometry, and capillary electrophoresis (CE).^[^ ^10^, ^12^, ^14^ ^]^ Among these, HPLC methods face difficulties associated with peak tailing and low efficacy. Requirements of large volumes of solvents, derivatizing treatment, and high capital investments are disadvantages as well. Chromatographic combination technologies chiefly employ the sensing techniques of mass spectrometry and chromatography coupled with other complicated procedures, although mature, but always demand big, immovable, and expensive instrumentation as well as skilled professionals. CE is not sensitive enough to determine low concentrations, because of the low sample‐injection volume and the short optical path length for on‐capillary detection. Thus, analyzing traces of residues in their low concentration is difficult in CE. In addition, these conventional analytical procedures are greatly limited by the complicated pretreatment process, and time‐consuming in analysis. In this context, luminescent sensing technology has the particular advantage of high efficiency, regenerability for multiple cycles, low cost, and ease of operation and is also widely adopted because of its environmental friendliness and economical characteristics.^[^ ^15^, ^16^, ^17^, ^18^, ^19^, ^20^, ^21^, ^22^, ^23^, ^24^ ^]^

Metal–Organic Frameworks (MOFs) having large surface area, highly porous structure, multi‐dimensionality, and structure adaptability have shown excellent luminescence sensing performance toward ultrahigh‐detection of harmful analytes with high selectivity and high sensitivity.^[^ ^25^, ^26^, ^27^, ^28^, ^29^, ^30^, ^31^, ^32^, ^33^, ^34^, ^35^, ^36^, ^37^, ^38^ ^]^ In this regard, d^10^ metal centres (Zn^2+^ and Cd^2+^) coupled with fluorogenic linkers containing electron rich π‐conjugated moieties are potentially strong candidates for designing multi‐functional luminescent MOF (LMOF) materials.^[^ ^39^, ^40^, ^41^, ^42^ ^]^ 2,6‐Naphthalenedicarboxylic acid (2,6‐H_2_NDC) is one such fluorogenic linker rich with photophysical and photochemical properties. Thus, MOFs constructed from 2,6‐H_2_NDC chromophores and their derivatives have shown broad prospects in the field of molecular luminescent probes.^[^ ^43^ ^]^ Meanwhile, owing to the closed shell electron configuration, d^10^ metal ions have very little interference to the charge transfer of the coordinated organic linkers, hence they become potential central metal ion candidates for the preparation of luminescent frameworks. Besides, MOFs having a multi‐responsive nature is another crucial attribute for practical applications, as they enhance the multipurpose usage possibility of the developed probes.^[^ ^39^ ^]^ Moreover, 2D MOFs possess a number of features that make them ideal for use in sensing applications. Unlike their 3D counterparts, the surface active sites in 2D MOFs are easily accessible to analytes allowing for more rapid diffusion and close contact between the targeted analytes and the framework, thus inducing a better interaction between them. Consequently, the employed sensor can exhibit an ultrahigh sensitive detection with reduced limits of detection of the toxic analytes compared to 3D MOF sensors, however, a greater selectivity could be achieved for 3D MOFs considering size of analytes and their fitting with pore windows.^[^ ^44^ ^]^ On the other hand, ultra‐detection of harmful analytes in an aqueous solution is preferred over a non‐aqueous medium owing to their affordability in real‐time device fabrication, which further emphasizes the aqueous medium stability of the employed frameworks.^[^ ^45^, ^46^, ^47^, ^48^, ^49^ ^]^ Thus the rational designing of MOFs employing mixed‐ligand (hard basic acid linkers and soft basic N‐donor linkers with hydrophobic moieties) approach is of paramount importance to ensure the chemical stability of the constructed framework. Furthermore, easy scalability is another important factor for real‐life applications.^[^ ^50^, ^51^, ^52^, ^53^ ^]^ Therefore, it is imperative to develop multi‐responsive luminescent 2D MOF sensors with high water stability and bulk scalability for the detection of toxic NFAs and OCPs.^[^ ^54^, ^55^, ^56^, ^57^, ^58^, ^59^, ^60^ ^]^

Thus, considering the following specific aspects, herein, we adopted a mixed‐ligand synthetic strategy to design a 2D robust Cd‐MOF, **IITKGP‐71** having molecular formula {[Cd(MBPz)(2,6‐NDC)]·2H_2_O}*_n_*: At first (i) redox‐inert Cd^2+^ with d^10^ configuration was chosen as metal centers, which is conducive to the efficient luminescence properties of **IITKGP‐71**; (ii) employing 2,6‐H_2_NDC acid ligand to impart hydrostability by hydrophobic shielding around the SBUs (*kinetic stability aspect*); (iii) the same 2,6‐H_2_NDC as a fluorogenic acid ligand with electron‐rich π‐conjugated system to be utilized for construction of LMOFs with notable luminescence emission abilities; (iv) usage of methylenebis(3,5‐dimethylpyrazole) (MBPz) spacer to form strong metal‐N bond (soft‐soft interaction, *thermodynamic stability aspect*) as well as dangling methyl moieties in imparting hydrophobicity (*kinetic stability aspect*) onto the framework to gain extra stabilization even in harsh conditions. Its bulk scalability and excellent structural stability further prompt us to investigate its luminescence properties toward organic pollutant detection in an aqueous solution. Importantly, the developed framework exhibited a selective and rapid luminescence quenching phenomenon for NFAs (NFZ and NFT) and DCN pesticide, offering high sensitivity and excellent recyclability. Combined with spectroscopic analysis and density functional theory calculations the luminescence sensing mechanism was established, showing that both resonance energy transfer (RET) and photo‐induced electron transfer (PET) are responsible for the exclusive quenching of NFAs and DCN with excellent detection ability and ultrafast kinetics.

## 2 Results and Discussion

**Scheme 1:**
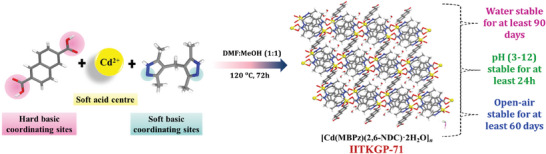
Illustration of the rationally designed and developed **IITKGP‐71**.

**Figure 1:**
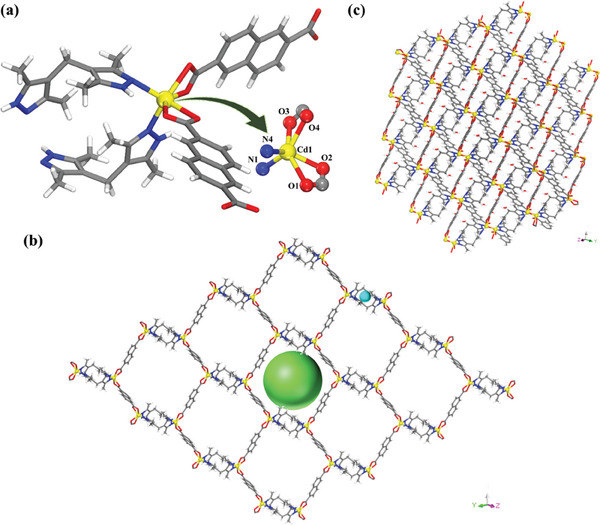
a) Coordination environment of Cd(II) in **IITKGP‐71**; b) one 2D layer fitted with two different coloured balls showing two different sized pore spaces with channel dimensions of 5.8 × 3.2 Å^2^ (small cyan ball) and 16 × 16 Å^2^ (large green ball), respectively; c) 2D packing diagram of **IITKGP‐71** (color code: Cd, yellow; O, red; C, gray; N, blue; H, white).

Pale yellowish block‐type single crystals of **IITKGP‐71** were afforded via the mixed‐ligand solvothermal reaction of cadmium(II) nitrate with MBPz spacer and 2,6‐H_2_NDC acid ligand (**Scheme** **1**) in a mixed DMF/MeOH solvent with a framework formula of {[Cd(MBPz)(2,6‐NDC)]·2H_2_O}*_n_*. SCXRD analysis revealed that **IITKGP‐71** crystallizes in triclinic crystal system with the space group of $P\bar{1}$ (Table S1, Supporting Information). The asymmetric unit contains one Cd^2+^ metal center, one full unit of the MBPz spacer, two half units of deprotonated 2,6‐NDC^2−^ acid ligands, and two lattice water molecules (Figure S1, Supporting Information). The detailed structural analysis showed that each Cd(II) center is six coordinated with four oxygen atoms {O(1), O(2), O(3), and O(4)} from two different acid units in bidentate coordinate mode and two nitrogen atoms {N(1) and N(4)} offered by two distinct pyrazole spacers, constructing distorted octahedral {Cd(COO)_2_N_2_} SBUs (**Figure** **1**a). The bond distances and bond angles around the central metal ion are within the normal range, as summarized in Table S2 (Supporting Information). The coordination of MBPz spacers with two such adjacent SBUs generates rhombus‐shaped binuclear {Cd_2_(MBPz)_2_} units having porous windows of 5.8 × 3.2 Å^2^ while considering the van der Waals radii (Figure 1b). Additionally, another two octahedral SBUs are connected with two dimeric units with the help of four acid linkers, thus leading to the formation of extended porous 2D layers with the dimensions of 16 × 16 Å^2^ (considering van der Waals radii into account) along the [111] direction (Figure 1b). Furthermore, these 2D layers are stacked upon each other and interact with each other via extensive H‐bonding, π⋯π stacking, and several non‐covalent interactions (Figure S2 and Table S3, Supporting Information) to build the overall 2D framework. Both the lattice water molecules are H‐bonded with each other; the H‐atom (H1W2) of one lattice water molecule OW(2) interacts with another lattice water molecule OW(1), while H2W1 of OW(1) forms H‐bonding interaction with the OW(2) atom (Figure S3 and Table S3, Supporting Information). The oxygen atom, O(3) of the acid ligand interacts with the hydrogen atom {H(2) attached with N(2)} of the pyrazole moiety from the same layer, while O(1) and O(4) oxygen atoms of the acid units form H‐bonding interaction with the hydrogen atom {H(3) attached with N(3)} of the pyrazole spacer from a different layer, thus connecting the two neighboring layers with stable H‐bonding interactions (Figure S3 and Table S3, Supporting Information). Additionally, the consecutive 2D stacked layers exhibit intermolecular π⋯π interactions between the pyrazole moieties of the spacers and benzene backbone of 2,6‐NDC^2−^ units with a distance of 3.681 Å (Figure S4, Supporting Information). Besides, several C‐H⋯O nonbonding interactions with a distance ranging from 2.70 to 3.42 Å are also present, which are summarized in Table S4 (Supporting Information). Such interactions play a vital role in making the framework chemically robust.

**Figure 2:**
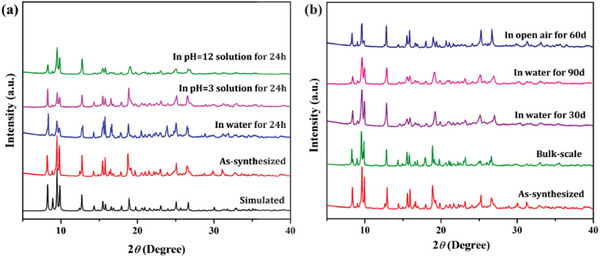
Comparative PXRD patterns of the a) as‐synthesized, 24 h water and aqueous pH (3 and 12) treated samples with that of the simulated pattern of **IITKGP‐71**; b) bulk‐synthesized, 60 and 90 days water immersed and open lab atmosphere treated samples compared with that of the as‐synthesized pattern of **IITKGP‐71**.

The PXRD pattern of the as‐synthesized material is in accordance with the simulated one, indicating the phase purity of the as‐synthesized sample (**Figure** **2**a). Besides phase‐purity, bulk scalability and high chemical stability assessments are two key points for the practical applicability of any developed framework in multifarious fields. Interestingly, the overnight refluxing of the reactants in the DMF/MeOH solvent mixture provided good‐quality tiny crystals of **IITKGP‐71**, implying easy scalability of the 2D MOF (SI, Experimental section). Furthermore, the excellent retention of PXRD patterns of the bulk sample with that of the as‐synthesized material confirms the phase purity of the bulk‐synthesized MOF (Figure 2a). The water and organic solvent stability along with the aqueous medium pH stability were verified by immersing the finely ground as‐synthesized sample in their respective solutions. The indistinguishable PXRD patterns after 24 h immersion in water, acidic (pH = 3), and basic (pH = 12) aqueous solutions, common laboratory solvents, and open‐air stability (even after exposure for two months) confirm the remarkable chemical robustness of the developed Cd‐MOF (Figure 2a,b; Figure S5, Supporting Information). The hydrostability of the framework was further extended up to several days, which exhibits the highly robust nature of the material even after 90 days of water immersion (Figure 2b) and further lays the foundation for luminescence sensing in water medium. Most importantly, we have also collected the SC‐XRD structure of **IITKGP‐71** after treating it in water for a prolonged time. Even after 30 days of water‐treatment the obtained single crystal structure didn't exhibit any significant change in crystallographic cell parameters, which further supports the excellent hydrostability of **IITKGP‐71** (Table S1, Supporting Information). Such a high chemical stability could be ascribed to the combined effect of the presence of stronger metal‐N coordination bonds (thermodynamic stability aspect), dangling hydrophobic naphthyl rings of the acid linker, and methyl functionalities of the spacer (kinetic stability aspect), H‐bonding interactions, π⋯π interactions, and C‐H⋯O non‐covalent interactions between the 2D stacked layers. TGA analysis of the as‐synthesized sample displays that the framework is stable up to 375 °C with an initial wt. loss of 7.09% (calc. 6.3%), which can be attributed to the loss of two lattice water molecules along with some moisture that gets adsorbed during the transfer of the sample to the alumina crucible (Figure S6, Supporting Information). FESEM analysis revealed block‐shaped surface morphology of **IITKGP‐71** while, EDX and elemental mapping analysis ensured a uniform distribution of all the desired elements (Cd, C, O, and N) within the whole framework, which further validates the purity of the as‐synthesized material (Figure S7, Supporting Information).

Given the excellent framework robustness, bulk scalability, presence of redox‐inert d^10^ metal centers, and large π‐electron conjugated system, the luminescence property of **IITKGP‐71** was explored for the detection of antibiotic and pesticide molecules in aqueous medium. The photoluminescence behavior for both **IITKGP‐71** and the free 2,6‐H_2_NDC organic linker was conducted in aqueous medium at room temperature environment. A strong emission peak at 369 nm (λ_ex_ = 280 nm) was observed for the developed LMOF, which mainly appears due to the corresponding intra‐ligand π → π* or n → π* electronic transitions of the π‐electron rich acid ligand. Moreover, the emission spectrum of **IITKGP‐71** was similar to that of the free 2,6‐H_2_NDC ligand (λ_ex_ = 284 nm, λ_em_ = 367 nm), which proved that the luminescence of the framework was completely ligand‐centered. Thus it could be speculated that the luminescence emission phenomenon of **IITKGP‐71** is mainly derived from the combined effect of the intra‐ligand charge transfer and ligand‐to‐ligand charge transfer (LLCT) within the proximity between nearest neighboring π‐conjugated acid linkers instead of metal‐to‐ligand charge transfer (MLCT) nor ligand‐to‐metal charge transfer (LMCT) owing to the redox‐inert d^10^ configured Cd^2+^ ion (Figure S8, Supporting Information).

**Figure 3:**
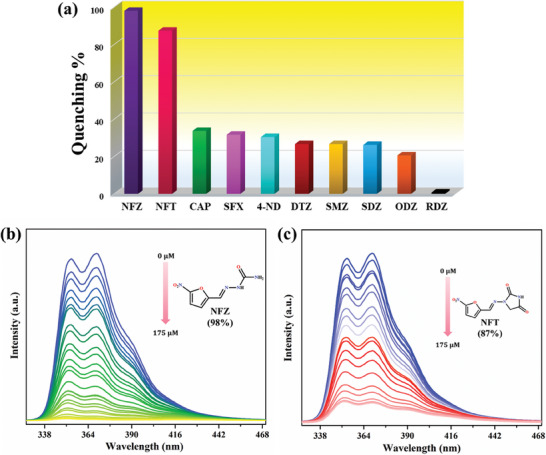
a) Selectivity of **IITKGP‐71** toward detection of different classes of antibiotics; emission spectra of **IITKGP‐71** upon the addition of 10^−3^ M aqueous solution of b) NFZ, and c) NFT.

After confirming the excellent luminescence properties of **IITKGP‐71**, its potentiality as a luminosensor material was assessed toward selective detection of different classes of toxic antibiotics {nitrofurans [nitrofurazone (NFZ) and nitrofurantoin (NFT)], chloramphenicol (CAP), nitroimidazoles [4‐Nitroimidazole (4‐ND), dimetridazole (DTZ), ornidazole (ODZ), and ronidazole (RDZ)], sulfonamides [sulfamethazine (SMZ), sulfadoxine (SFX), and sulfadiazine (SDZ)]} (Figure S9, Supporting Information). Initially, 200 µL of the analyte solution (10^−3^ M concentration) was gradually added (in the interval of 20 µL) to 2 mL of the dispersed MOF solution, and the corresponding emission intensity centered at 369 nm was monitored. The results showed that NFZ and NFT could exclusively elicit luminescence quenching of **IITKGP‐71**, while other antibiotics offered mild to no quenching effects on the corresponding luminescence intensity of the employed sensor probe (**Figure** **3**a). Such markedly different quenching effects of nitrofuran antibiotics (NFAs) toward the luminescence emission of **IITKGP‐71** reveal that **IITKGP‐71** can serve as a promising luminescent sensor for the detection of NFAs with high selectivity.

In order to verify the sensitivity of **IITKGP‐71** toward NFAs quenching, the quantitative detection for NFZ and NFT was investigated. As shown in Figure 3b,c, the emission intensities decreased gradually with the increasing volumes of NFAs, and around 98% quenching of initial luminescence intensity was obtained for NFZ analyte, whereas NFT exhibited 87% declination of intensity. Furthermore, the emission peak at 369 nm displayed a linear relationship at a lower concentration range of the NFAs with good correlation coefficient values of *R* ^2^ = 0.9948 (for NFZ) and 0.9912 (for NFT), respectively (Figure S10, Supporting Information). With a further increase in the concentration of the analytes, the Stern‐Volmer (S‐V) plot deviated from linearity, which could be ascribed to the presence of energy transfer processes or static or dynamic quenching processes. The quenching constant (K_sv_) values were obtained as 3.1 × 10^4^ and 2.1 × 10^4^ m ^−1^ for NFZ and NFT, respectively from the corresponding S‐V plot, which shows higher effectivity for NFZ antibiotic between the two NFAs. As the key evaluating index for sensitivity, the limit of detection (LOD) was calculated from 3σ/K_sv_ (σ = standard deviation) and found to be as low as 0.11 and 0.17 µm for NFZ and NFT, respectively, which again established the superior sensitivity of **IITKGP‐71** for nitrofuran class of antibiotics. In addition, the luminescence quenching efficiency of **IITKGP‐71** to NFZ and NFT in water was recorded at different time intervals with different concentrations of analytes. In all cases, the *turn‐off* sensing phenomenon takes place within a few seconds, indicating the rapid response capability of **IITKGP‐71** toward this direction (Figure S11, Supporting Information).

**Figure 4:**
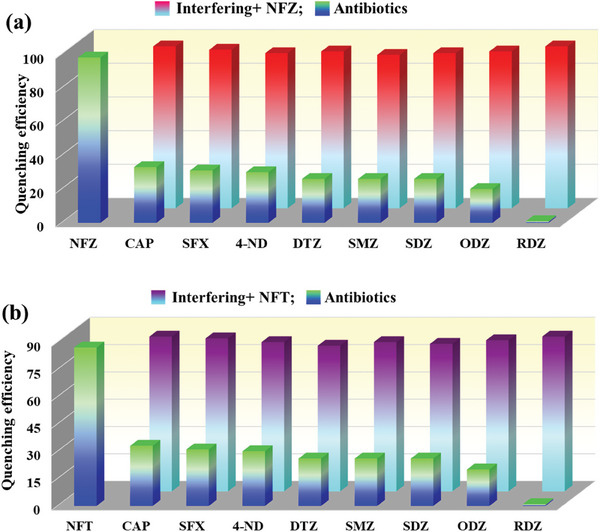
Anti‐interference test of **IITKGP‐71** for a) NFZ and b) NFT with other classes of antibiotics, respectively.

Furthermore, the highly sensitive and selective sensing of NFAs prompted us to assess the anti‐interference ability for the ultra‐detection of NFZ and NFT in the presence of other classes of antibiotics. The aqueous solution of other interfering antibiotics was mixed with and without the NFAs in the dispersed solution of MOF samples and their luminescence were recorded at room temperature. The luminescence of **IITKGP‐71** was observably quenched when NFAs were introduced into the mixed antibiotic compounds (**Figure** **4**), affirming that the MOF sensor can effectively detect the toxic NFAs and the detection was not affected by other coexisting classes of antibiotics. Therefore, **IITKGP‐71** can potentially serve as a smart luminescent probe for monitoring NFAs in the aqueous medium with great selectivity, high sensitivity, rapid responsiveness, and excellent anti‐interference capability.

In practice, the sensor should have the ability to be applied for the recognition of desired contaminants with long‐term durability and repeatability. It should also be noted that even after six consecutive cycles (in a day) the quenching efficiencies and structural integrity for NFZ and NFT were retained, which highlights the excellent recyclability of the framework toward this projected application (Figure S12a–c, Supporting Information). To verify the long‐term stability we immersed as‐synthesized material, **IITKGP‐71**, in the NFZ, and NFT analyte solutions separately for two weeks and collected the PXRD patterns. Interestingly, in all the cases, PXRD patterns are in well‐agreement with the as‐synthesized one, which confirms the excellent stability of **IITKGP‐71** even in the presence of targeted analytes for a prolonged time (Figure S13a, Supporting Information). Moreover, to find out the long‐term continuous detection and performance changes if any, we have performed the luminescence sensing experiment for three weeks at the intervals of 3 or 4 days, with consecutive recycled MOF sample, in which, no significant change in the quenching performance was obtained even after reaching the equilibrium in the first day of experiment (Figure S13b, Supporting Information). All of these studies evidence the long‐term stability and reliability of the developed sensor material, **IITKGP‐71**.

Furthermore, we have assessed the potentiality of **IITKGP‐71** toward aqueous medium sensing of NFAs by comparing its performance with the previously reported luminescent MOFs/CPs (Table S5, Supporting Information). It is worth noting that the K_sv_ values of our work are better than or comparable with reported values while the detection limits of **IITKGP‐71** are among the lowest LODs in comparison with those framework‐based luminosensors for NFAs detection (Table S5, Supporting Information). This analysis showcased the suitability of the developed probe in this direction. Importantly, the majority of NFAs detection experiments for MOF and/or CPs‐based sensors were carried out in non‐aqueous solvents rather than water owing to their chemical instability in aqueous medium, making them less useful in realistic environments. However, **IITKGP‐71** upholds a relatively better *trade‐off* between a higher K_sv_ and lower LOD for aqueous medium detection of toxic nitrofuran antibiotics, which again highlights its practical efficiency toward this projected application.

**Figure 5:**
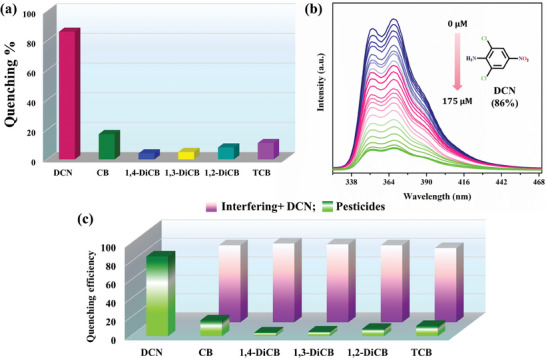
a) Selectivity of **IITKGP‐71** toward pesticides sensing; b) luminescence spectra of **IITKGP‐71** upon gradual addition of 10^−3^ M solution of DCN; c) anti‐interference test for DCN with other pesticide analytes.

In order to extend the applications of **IITKGP‐71** in optical sensing, its luminescence detection performance was further investigated similarly toward several pesticides including 2,6‐dichloro‐4‐nitroaniline (DCN), chlorobenzene (CB), 1,2‐dichlorobenzene (1,2‐DiCB), 1,4‐dichlorobenzene (1,4‐DiCB), 1,3‐dichlorobenzene (1,3‐DiCB), and 1,2,4‐trichlorobenzene (TCB) (Figure S14, Supporting Information). As shown in **Figure** **5**a, when 200 µL of the pesticide analytes were qualitatively added into the dispersed solutions of the **IITKGP‐71**, only DCN exhibited significant luminescence quenching effects, while no apparent quenching was found for other analytes. These findings demonstrated that **IITKGP‐71** exhibited excellent selectivity for DCN detection in aqueous medium.

To figure out the quantitative relationship between the pesticide contents and the emission intensity of **IITKGP‐71**, one series of luminescence titration operations was performed. Concentration‐dependent luminescence spectra presented in Figure 5b indicated that by gradually increasing the concentration of DCN, the initial emission intensity was progressively decreased and a maximum quenching of ∼86% was obtained. More strikingly, the quenching efficiency illustrated a good linear dependency with the lower concentrations of DCN, which was precisely simulated using the S‐V equation, and the corresponding K_sv_ value was obtained as 1.6 × 10^4^ M^−1^ with R^2^ value of 0.9924 (Figure S15, Supporting Information). From the 3σ/K_sv_ ratio, the LOD value was calculated, and the developed MOF displayed a significantly lower LOD value of 0.21 µm, indicating high sensitivity of **IITKGP‐71** toward DCN sensing. Moreover, aside from retaining a high selectivity and high sensitivity, the developed sensory material needs to be fast enough for quick on‐site detection of the targeted toxic analytes; then, only it could be utilized in practical applications. The luminescence kinetic study showed that the developed MOF can sense DCN within a few seconds of the addition of the targeted analyte to the dispersed fluorophore suspension, which again highlighted the fast‐responsive nature of the MOF in this direction (Figure S16a, Supporting Information).

Besides, the effective selectivity and excellent sensitivity of **IITKGP‐71**, the anti‐interference property is another crucial factor in deciding its suitability for real‐world application. The competitive luminescence experiments with the equivalent amount of other pesticides displayed that the emission intensity of **IITKGP‐71** could still be significantly quenched even in the presence of existing interfering analytes (Figure 5c). The recyclability experiment exhibits no significant performance loss even after six successive measurements (in a day), which highlights the excellent recyclability of the material for the detection of DCN (Figure S16b, Supporting Information). Furthermore, the sensor material can retain its structural integrity even after 15 days of immersion in targeted DCN analyte (Figure S13a, Supporting Information). Again, no significant loss of quenching efficiencies for three weeks at an interval of 3 or 4 days, with consecutive recycled MOF sample could be observed, as displayed in Figure S13b (Supporting Information). The stability of the framework following DCN sensing can be verified by the unaltered PXRD pattern with that of the as‐synthesized one (Figure S12c, Supporting Information). The outstanding anti‐interference capability along with multi‐cycle reusability suggests that **IITKGP‐71** can act as a smart luminescence sensor for DCN pesticide determination with excellent specificity and high sensitivity.

Additionally, the sensing performance of **IITKGP‐71** toward DCN organochlorine pesticide detection was compared with the previously reported luminescent MOFs/CPs (Table S6, Supporting Information). In most of the cases, as listed in Table S6 (Supporting Information), the sensing experiments are performed in non‐aqueous medium (DCM, EtOH, DMF, and DMA), simply because of their chemical instability in water medium, which is indeed necessary in terms of regularity and practicality. Interestingly, **IITKGP‐71** showed one of the lowest LOD values for aqueous medium DCN sensing as compared to other water‐stable MOF/CP‐based probes, which proves the superiority of the constructed MOF for this projected application.

To verify the effectivity of MOF sensor over bared fluorogenic organic ligand (2,6‐H_2_NDC), we performed the luminescent quenching study with 2,6‐H_2_NDC in aqueous medium, similar to the aqueous medium MOF dispersion. Significantly lower quenching efficiencies (46, 40 and 37% for NFZ, NFT, and DCN analytes, respectively, Figure S17, Supporting Information) were observed for the ligand only, whereas the MOF, **IITKGP‐71** exhibited the maximum corresponding quenching efficiencies of 98, 87 and 86% for NFZ, NFT, and DCN analytes, respectively. Such an enhanced quenching performance for the developed MOF over the ligand only could be attributed to the higher conjugated structure formation (with the association of electron‐rich metal center and employed ligand) and hindered the free rotation of the ligand. Hence, this comparative study demonstrates the necessity and effectivity of MOF to be used as a sensor material rather than the ligand only.

**Figure 6:**
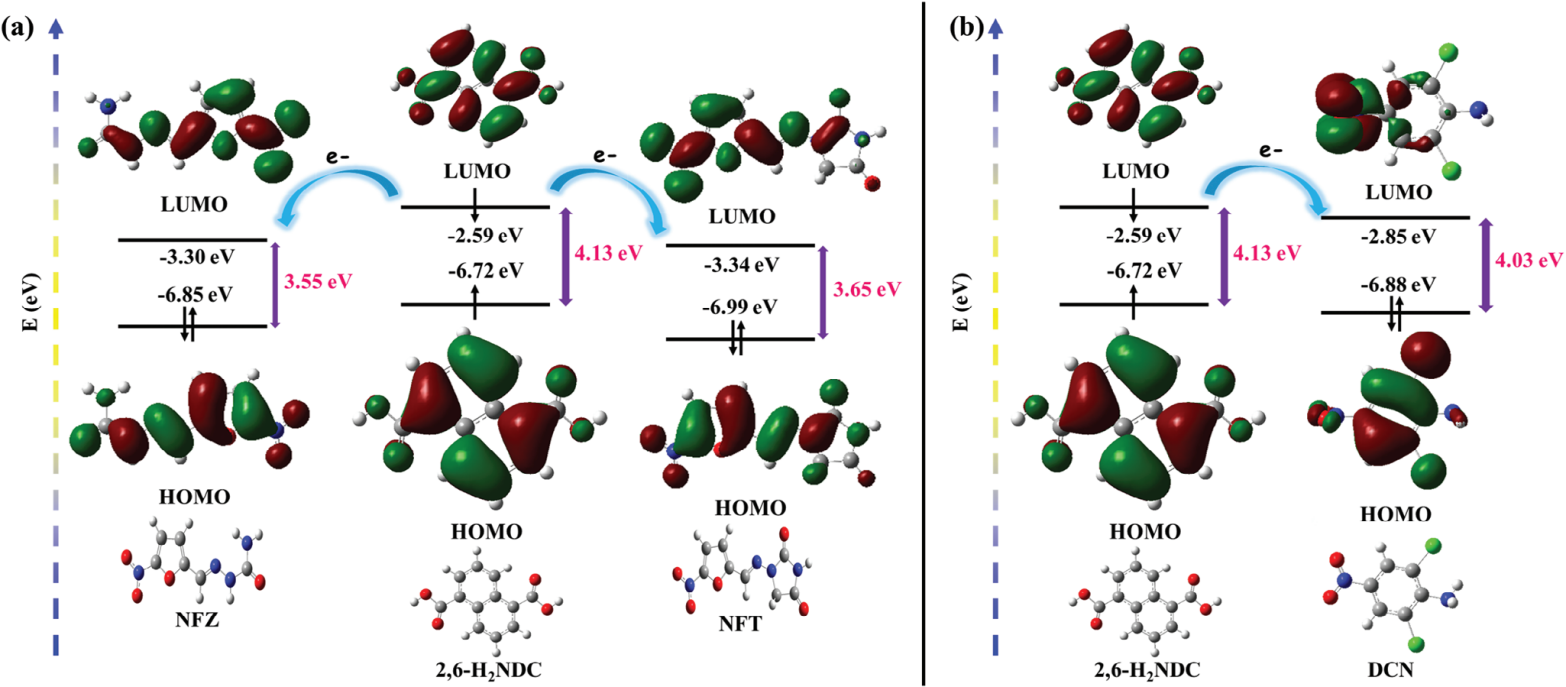
Comparison of HOMO−LUMO energy levels of 2,6‐H_2_NDC with a) NFZ and NFT antibiotics and b) DCN organochlorine pesticide showing facilitated PET process.

To elucidate the mechanism underlying the aforementioned quenching effects, further investigations have been conducted. First, the possibility of luminescence quenching induced by structural collapse is ruled out due to the unaltered PXRD pattern and IR spectra of MOF **IITKGP‐71** even after six detection cycles (Figure S12c,d, Supporting Information). Secondly, after adding different concentrations of NFZ, NFT, or DCN, the luminescence lifetime of **IITKGP‐71** remains almost unchanged, which suggests the absence of any strong interactions between the guest analytes and the host framework skeleton (Figure S18, Supporting Information). Besides, the fast kinetics with easy regeneration of the probe indicates that the luminescence quenching is not due to guest molecule adsorption. Moreover, for a better understanding of the preferential luminescence quenching effect instigated by NFAs and OCPs, the UV─vis spectrum of the NFAs and DCN along with other interfering analytes was recorded in aqueous media, which displays significant spectral overlapping of NFAs’ and DCN's characteristic UV─vis band with the emission spectrum of **IITKGP‐71** (Figures S19 and S20, Supporting Information). As for the other antibiotics and pesticides except NFAs and DCN, the emission spectra are non‐overlapping with that of developed MOF, indicating that there is negligible detection effects on other analytes. This finding confirms that resonance energy transfer (RET) occurs between the excited state of the luminescent probe and the ground state of NFAs and DCN, which is largely accountable for the remarkable quenching in the emission of **IITKGP‐71**. Other than the RET mechanism, the photo‐induced electron transfer (PET) phenomenon is another recognized quenching process, involving electron transfer from the luminescent probe's LUMO to the LUMOs of the analytes. Thus, the effective detection of the targeted analytes was further rationalized by density functional theory (DFT) calculation of the energy bands of the antibiotics and pesticides, which revealed that the HOMO‐LUMO energy values of the acid ligand perfectly corroborate with HOMO‐LUMO energy parameters of NFZ, NFT, and DCN (**Figure** **6**). Hence, it can be concluded that the PET mechanism additionally contributes to the reduction in emissive response during the sensing of NFAs and DCN. Moreover, the LUMO energy levels of the antibiotics are in the order NFT < NFZ < CAP, RDZ < DTZ < ODZ < 4‐ND < SDZ < SMZ < SFX, whereas in the case of pesticides the LUMO levels are in the order of DCN < TCB < 1,4‐DiCB < 1,3‐DiCB < 1,2‐DiCB < CB (Figures S21 and S22, Supporting Information). It is known that the lower the LUMO energy of the analytes, the more easily the electrons are transferred from electron‐rich framework structure of MOFs to the electron‐deficient analytes. Thus, although some of the antibiotics can quench the luminescence of **IITKGP‐71** to a certain extent, except for RDZ, the luminescence quenching phenomenon is the most significant in NFAs (NFT and NFZ) while guaranteeing structural stability. Notably, the LUMO energy level of RDZ is closest to CAP in this series of antibiotics, which however shows almost no quenching effect. This reveals that electron transfer is not the sole contributing factor for luminescence quenching. Thus, the luminescence quenching of the developed sensor for toxic NFA antibiotics and DCN pesticide sensing is mainly based on the RET mechanism along with a little contribution from the PET process.

Hence, it can be inferred that the **IITKGP‐71** exhibited exceptional luminescence properties for the ultra‐sensitive detection of toxic organic pollutants (NFZ, NFT, and DCN) in aqueous medium with excellent sensitivity, high selectivity and outstanding reusability. The chemical robustness coupled with bulk scalability further emphasizes the real‐time applicability of the developed framework as a multi‐responsive luminosensor in the wake of nitrofuran antibiotics and organochlorine pesticide abuse.

## 3 Conclusion

In summary, employing the mixed‐ligand synthetic approach, we strategically designed and developed an ultra‐stable 2D Cd‐MOF (**IITKGP‐71**), which acts as a dual sensor for the detection of toxic NFAs and DCN in the aqueous medium. The developed framework displayed excellent chemical stability in water for three months and can resist acids or bases over a wide pH range from 3 to 12. With the synergistic effect of the RET mechanism and the PET process, **IITKGP‐71** exhibited high quenching efficiency with excellent selectivity, ultrahigh sensitivity, rapid responsivity, and multiple recyclabilities for the targeted organic pollutants. The multi‐responsive *turn‐off* sensing phenomenon along with easy scalability and high chemical stability undoubtedly demonstrate that **IITKGP‐71** is viable for fabricating apt luminosensors with promising application prospects. Thus, this study emphasized the importance of chemically stable luminescent frameworks for inducing trace detection of toxic organic pollutants and also offered a solid approach and theoretical backing for the subsequent MOF sensor design in the context of ensuring environmental protection and public security.

## Conflict of Interest

The authors declare no conflict of interest.

## Supporting information



Supporting Information

## Data Availability

The data that support the findings of this study are available in the supplementary material of this article.
