# Supplementary material for: Highly Water‐Stable 2D MOF as Dual Sensor for the Ultra‐Sensitive Aqueous Phase Detection of Nitrofuran Antibiotics and Organochlorine Pesticides
Source: Small. 2024 Nov 20;21(3):2409095. doi: 10.1002/smll.202409095 (PMC11753491; doi:10.1002/smll.202409095)
Supplement: Supplementary file 1 — Supporting Information [file SMLL-21-2409095-s001.docx]

**Supporting Information (SI)**

**Highly Water-Stable 2D MOF as Dual Sensor for the Ultra-Sensitive Aqueous Phase Detection of Nitrofuran Antibiotics and Organochlorine Pesticides**

Supriya Mondal, Rupam Sahoo and Madhab C. Das*

*Department of Chemistry, Indian Institute of Technology Kharagpur, Kharagpur 721302, WB, India.*

*E-mail:* [*mcdas@chem.iitkgp.ac.in*](mailto:mcdas@chem.iitkgp.ac.in)

**Table of Contents**

|  | Materials and Methods | S2-S3 |
| --- | --- | --- |
|  | Experimental section and sensing experiments | S3-S5 |
| **Figures S1-S4** | Asymmetric unit, 2D stacked layers, H-bonding, π-π and interactions of **IITKGP-71** | S5-S6 |
| **Figure S5** | Solvent stability of **IITKGP-71** as verified by PXRD analysis | S7 |
| **Figure S6** | TGA plot of as-synthesized **IITKGP-71** | S7 |
| **Figure S7** | FESEM images, EDX and elemental mapping of **IITKGP-71** | S7 |
| **Figure S8** | Comparison of emission spectrum of **IITKGP-71** and 2,6-H_2_NDC in solution state, and standard deviation plot | S8 |
| **Figure S9** | Optimized structures of the antibiotic analytes | S8 |
| **Figure S10** | Stern−Volmer plots for NFZ, NFT sensing | S9 |
| **Figure S11** | Kinetic plots for NFZ, NFT sensing | S9 |
| **Figure S12** | Recyclability plots for NFZ, NFT sensing and PXRD, IR of **IITKGP-71** after NFZ, NFT, DCN sensing | S10 |
| **Figure S13** | Long term stability and luminescence quenching study plot for NFZ, NFT, DCN sensing | S10 |
| **Figure S14** | Optimized structures of the pesticide analytes | S11 |
| **Figure S15** | Stern−Volmer plot for DCN sensing | S11 |
| **Figure S16** | Kinetic and recyclability plots for DCN sensing | S12 |
| **Figure S17** | Comparison of PL performance between **IITKGP-71** and **2,6-H_2_NDC** | S12 |
| **Figure S18** | Lifetime decay profile of **IITKGP-71** before and after the addition of the NFZ, NFT and DCN solutions, respectively | S13 |
| **Figures S19-S20** | Spectral overlaps between the UV-vis absorption spectrum of different antibiotic and pesticide analytes with the emission spectra of **IITKGP-71**, respectively | S14 |
| **Figures S21-S22** | Comparison of HOMO-LUMO orbitals of the interfering antibiotics and pesticides with that of 2,6-H_2_NDC linker, respectively | S15 |
| **Table S1** | Crystallographic Data of **IITKGP-71** and **IITKGP-71w** | S16 |
| **Table S2** | Selected Bond Distances (Å) and Bond Angles (º) in **IITKGP-71** | S16-17 |
| **Tables S3-S4** | H-bonding and other non-bonding interactions in **IITKGP-71** | S17 |
| **Tables S5-S6** | Comparison table of K_sv_ and LOD of **IITKGP-71** towards NFZ, NFT and DCN with other CP/MOFs | S17-S19 |
|  | References | S19 |

**Physical Measurements.** The single-crystal XRD diffraction data were collected at 296 K on a Bruker AXS (D8 Quest System) X-ray diffractometer, equipped with a PHOTON 100 CMOS detector using graphite-monochromated Mo-K*_α_* radiation (0.71073 Å). PXRD patterns were recorded using *Cu-K_α_* radiation (1.5418 Å) on a Bruker D8 Advance diffractometer. Thermogravimetric analysis (TGA) was performed using a TG 209 *F3* Tarsus (Netzsch), and the sample was heated from room temperature to 800 °C at a rate of 10 °C min^-1^ under N_2_ atmosphere. The morphology and elemental analysis were examined on a Carl Zeiss MERLIN field emission scanning electron microscopy (FESEM) and ZEISS EVO 60 Scanning Electron Microscope equipped with Oxford energy dispersive X-ray spectroscopy (EDX) Detector. The IR spectra were recorded in the range of 400-4000 cm^-1^ on a Perkin-Elmer RX1 spectrophotometer. The luminescence spectra for the solution sample were recorded using a Shimadzu-RF-6000 spectrophotometer. The UV−Vis spectra for the analyte solutions were measured at room temperature using a Shimadzu UV-2600 UV-Vis spectrophotometer. Time-correlated single photon counting (TCSPC) spectrometer of IBH (U.K.) was used for the lifetime measurements.

**Computational method.** The energy level of the employed acid ligand and the analytes were calculated using Gaussian-16 software packages. The ground states of the molecules were optimized using the density function theory (DFT) exchange-correlation function B3LYP with basis set of 6-311++G**.

**Single Crystal X-ray Diffraction.** Good quality single crystals of **IITKGP-71** and **IITKGP-71w** were sorted out with the help of a polarizing microscope and immersed in paratone oil, which was then mounted on the tip of glass fiber and cemented using epoxy resin. The single-crystal XRD diffraction data were collected at 296 K and 120 K, respectively on a Bruker AXS (D8 Quest System) X-ray diffractometer, equipped with a PHOTON 100 CMOS detector using graphite-monochromated Mo-K*_α_* radiation (0.71073 Å). The linear absorption coefficients, scattering factors for the atoms, and the anomalous dispersion corrections were taken from International Tables for X-ray Crystallography. Bruker Apex III software was used for data collection, unit cell measurements, absorption corrections, scaling, and integration.^1,2^ The data were reduced and an empirical absorption correction was applied with the help of SAINTPLUS software and SADABS programs using XPREP, respectively.^3,4^ The structures were solved by the direct method using SHELXL-2014 in the WinGx programs. The WinGx package of programs was used to carry out the full-matrix least-squares refinement against the function |*F^2^*|.^5,6^ For all the cases, non-hydrogen atoms were refined anisotropically. The hydrogen atoms on both the linkers were geometrically fixed using the riding atom model and assigned fixed isotropic displacement parameters, whereas, the hydrogen atoms of lattice water molecules are assigned from electron density map. The “ACTA” command was used to generate the Crystallographic Information File (CIF). The structural details of **IITKGP-71** and **IITKGP-71w** are presented in **Table S1**. CCDC: **2388546** and **2401218** contains the crystallographic data for **IITKGP-71** and **IITKGP-71w**, respectively. These datas are available from The Cambridge Crystallographic Data Center (CCDC) *via* [www.ccdc.cam.ac.uk/data_request/cif](http://www.ccdc.cam.ac.uk/data_request/cif).

**Materials and reagents.** Cd(NO_3_)_2_·6H_2_O (Merck), 2,6-Naphthalenedicarboxylic acid (Alfa Aesar), hydrazine hydrate (Merck), acetylacetone (Spectrochem), formaldehyde (SRL), ethyl acetate (Merck), Na_2_SO_4_ (Spectrochem), Antibiotics (BLD), DCN (sigma) and other Chloropesticides (SRL and Spectrochem) and organic solvents were used as obtained, without further purification. The methylenebis(3,5-dimethylpyrazole) (MPBz) spacer was prepared according to the previous literature method.^7^

**Synthesis of IITKGP-71.** A mixture of Cd(NO_3_)_2_.6H_2_O (15 mg, 0.05 mmol), 2,6-H_2_NDC (11 mg, 0.05 mmol), and MBPz (10 mg, 0.05 mmol) was taken together in 8 ml mixed solvent of DMF/MeOH (1:1 v/v) and stirred at room temperature until the reactants are dissolved. The reaction mixture was then heated at 120 ºC for 72h in a sealed autoclave. Pale yellow colored block-shaped crystals were collected by filtration which was then analyzed *via* single crystal X-ray diffraction. Yield: ~80% (based on metal salt). Elemental analysis, Calcd: C_23_H_26_CdN_4_O_6_: C, 48.69%; H, 4.58%; N, 9.87%. Found: C, 49.51%; H, 4.53%; N, 9.15%.

***Bulk-scale* Synthesis of IITKGP-71.** A mixture of Cd(NO_3_)_2_·6H_2_O (1 mmol, 0.310 g), 2,6-H_2_NDC (1 mmol, 0.216 g), and MBPz (1 mmol, 0.200 g) were dissolved in 60 ml DMF/MeOH and refluxed in 120 ºC for overnight. After that, the crystals were filtered and washed with DMF three times to get the bulk amount of **IITKGP-71**.

**Sensing Experiments:** The PXRD analysis confirmed the excellent hydro-stability of **IITKGP-71**. Thus, all the sensing experiments were performed in aqueous medium. The standard solution was prepared by adding a finely ground sample of **IITKGP-71** (1 mg) in 2 mL water and treated by ultrasonication for about 30 min. Four different classes of antibiotics, nitrofurans [nitrofurazone (NFZ) and nitrofurantoin (NFT)], chloramphenicol (CAP), sulfonamide based antibiotics [sulfamethazine (SMZ), sulfadoxine (SFX), and sulfadiazine (SDZ)], and nitroimidazole based antibiotics [4-Nitroimidazole (4-ND), dimetridazole (DTZ), ornidazole (ODZ), and ronidazole (RDZ)] with 1 × 10^-3^ M concentration were prepared in water. Meanwhile, acetonitrile solutions of the organochlorine pesticides including 2,6-dichloro-4-nitroaniline (DCN), chlorobenzene (CB), 1,2-dichlorobenzene (1,2-DiCB), 1,4-dichlorobenzene (1,4-DiCB), 1,3-dichlorobenzene (1,3-DiCB), and 1,2,4-trichlorobenzene (TCB) were prepared with a concentration of 1 × 10^-3^ M. For the luminescent-based titration experiments 2 mL dispersed solution of **IITKGP-71** was taken in the cuvette with the gradual addition of the stock solution of analytes and the corresponding PL intensity was recorded. For all the experiments the excitation wavelength was 280 nm. To verify the effectivity of MOF sensing over bared employed fluorogenic organic ligand (2,6-H_2_NDC), the luminescent quenching study was performed in aqueous medium by dispersing 1 mg ligand in 2 ml water, similar to the aqueous medium MOF dispersion.

**Stern-Volmer quenching constant (K_sv_) and LOD calculations for NFZ, NFT and DCN:**

From the stock solution, the antibiotic and pesticide analytes were successively added to the blank dispersed MOF suspension, and luminescence spectra were recorded until the complete luminescence quenching was observed. To get the quantitative estimation of the quenching efficiency, the individual luminescence titration profiles were fitted to the Stern–Volmer equation, *I_0_/I = K_sv_[M] + 1*; where *I_0_* and *I* denote the luminescence intensity of the water dispersed solution of **IITKGP-71** before and after the addition of the stock solution of the NFZ, NFT and DCN, K_sv_ (M^–1^) and [M] implies the concentration of the added analytes and quenching constant, respectively. The fitting was performed at a lower concentration, which displayed an excellent linear relationship with the linear fit correlation coefficient of 0.9948, 0.9912 and 0.9924, respectively.

Considering the standard deviation (*σ*) *via* repeated luminescence measurements of the blank MOF solutions and based on the K_sv_ value, LOD values were calculated by the ratio of 3*σ*/*K_sv_*.

Photoluminescence (PL) spectra of blank solution of **IITKGP-71** with multiple measurements.

PL Intensities for blank solutions: 806088, 805142, 803186, 802799, 802843, 803744, 803241

Calculated standard deviation, *σ* = 1172.2 (**Figure S8b**)

**Competing antibiotics and pesticides selectivity studies:**

Anti-interference performance for NFT and NFZ was studied *by* taking an equimolar ratio of the nitrofurans with other competing classes of antbiotics [chloramphenicol (CAP), sulfamethazine (SMZ), sulfadoxine (SFX), dimetridazole (DTZ), ornidazole (ODZ), 4-Nitroimidazole (4-ND), ronidazole (RDZ) and sulfadiazine (SDZ)] and for DCN with other pesticides {chlorobenzene (CB), 1,2-dichlorobenzene (1,2-DiCB), 1,4-dichlorobenzene (1,4-DiCB), 1,3-dichlorobenzene (1,3-DiCB), and 1,2,4-trichlorobenzene (TCB)} in 2 mL dispersed solution of **IITKGP-71** followed by recording the corresponding emission intensity of the resultant solution.

**Quenching efficiency calculation:**

The maximum quenching efficiency was calculated using (1 − *I*/*I_0_*) × 100% equation in which *I* and *I_0_* denote the recorded emission intensities after and before the analyte addition, respectively.

**Recycling process:**

The reusability of the luminescence sensing performance of **IITKGP-71** toward the targeted analytes was examined for up to six cycles for NFAs and DCN. For checking the recyclability, **IITKGP-71** was centrifuged after each luminescence titration experiment followed by washing with water and acetone repeatedly and then dried in air. The probe showed outstanding recovery of the initial luminescence intensity, even after six consecutive cycles of luminescence sensing experiments. As verified by the PXRD experiment, the MOF material retained the initial crystallinity after six successive cycles for each luminescence titration experiment.


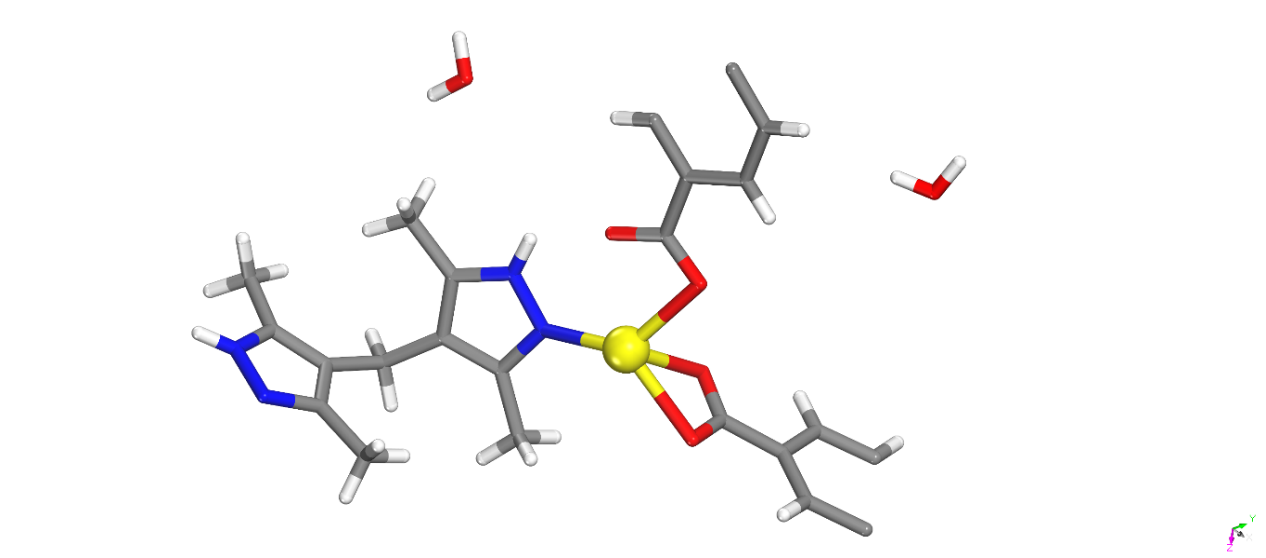


**Figure S1**: Asymmetric unit of **IITKGP-71** (color code: Cd, Yellow; O, red; N, blue; C, grey; H, white).


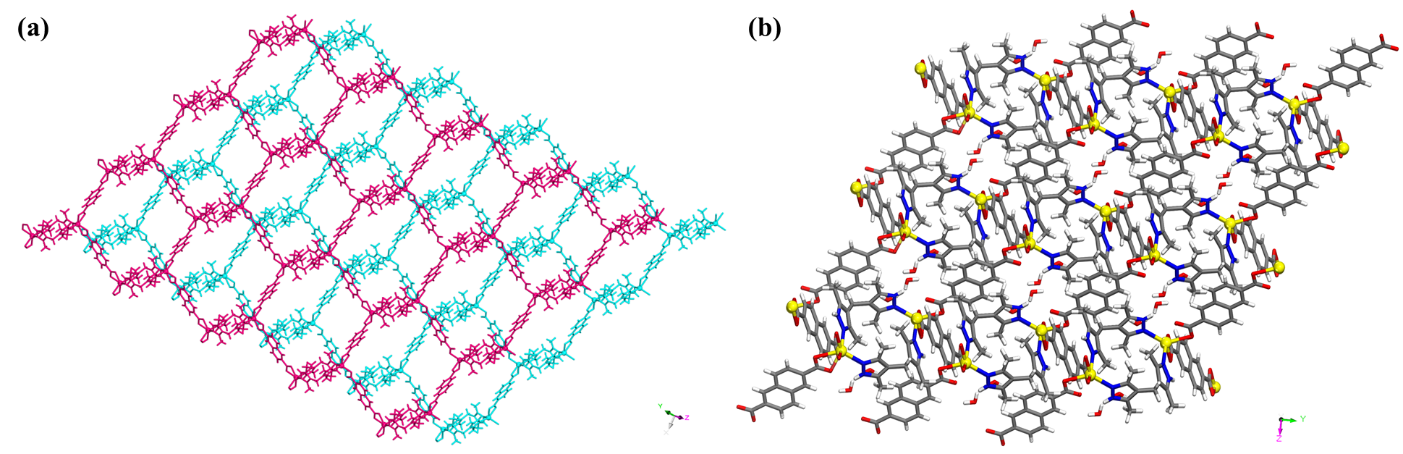


**Figure S2**: (a) Stacked 2D layers of **IITKGP-71** shown in two different colours; (b) Packing diagram along the crystallographic *a*-axis (color code: Cd, Yellow; O, red; N, blue; C, grey; H, white).


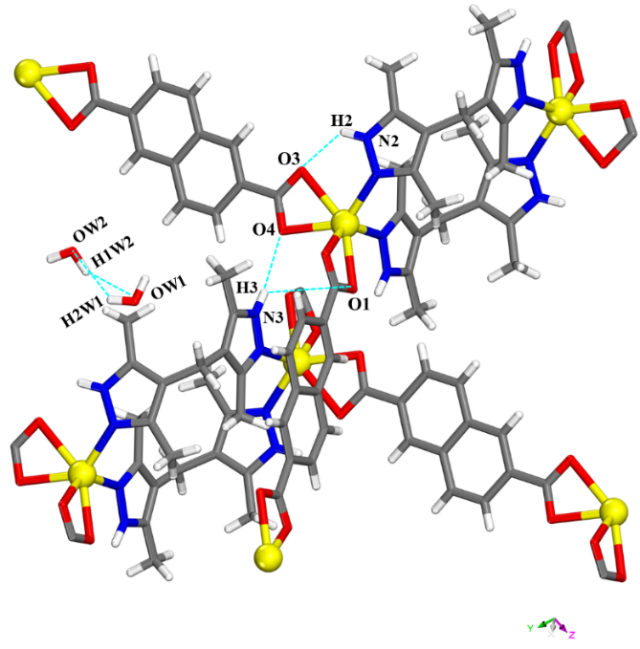


**Figure S3**: Extended H-bonding interaction between the adjacent 2D layers in **IITKGP-71** (color code: Cd, Yellow; O, red; N, blue; C, grey; H, white; H-bonds are shown in cyan colors).


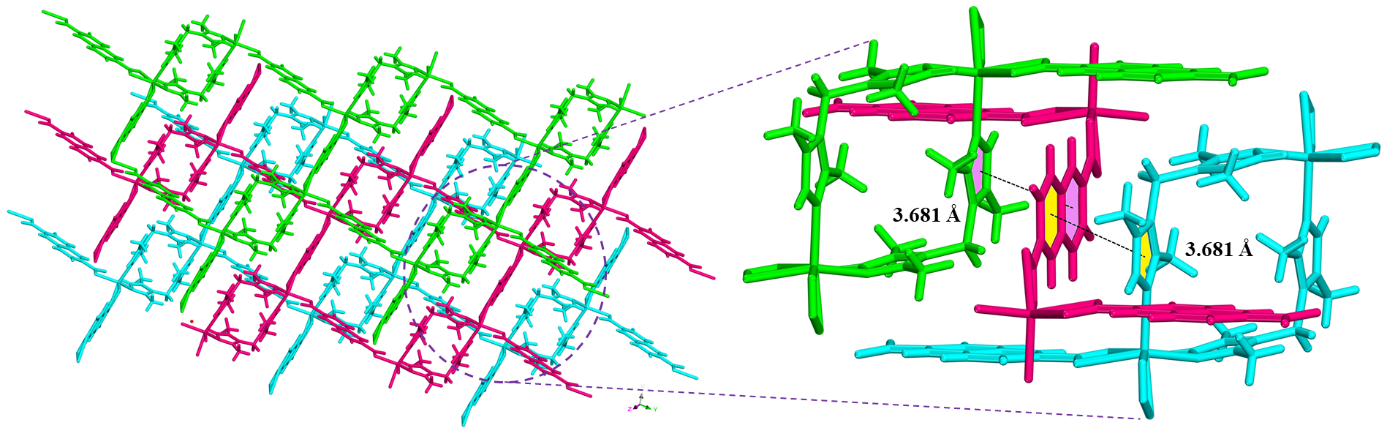


**Figure S4**: π⋯π interactions between the adjacent pyrazole moieties of spacer MBPz and phenyl ring of the 2,6-NDC^2-^ units in neighbouring layers with the distance of 3.681 Å in **IITKGP-71** (color code: Cd, Yellow; O, red; N, blue; C, grey; H, white).


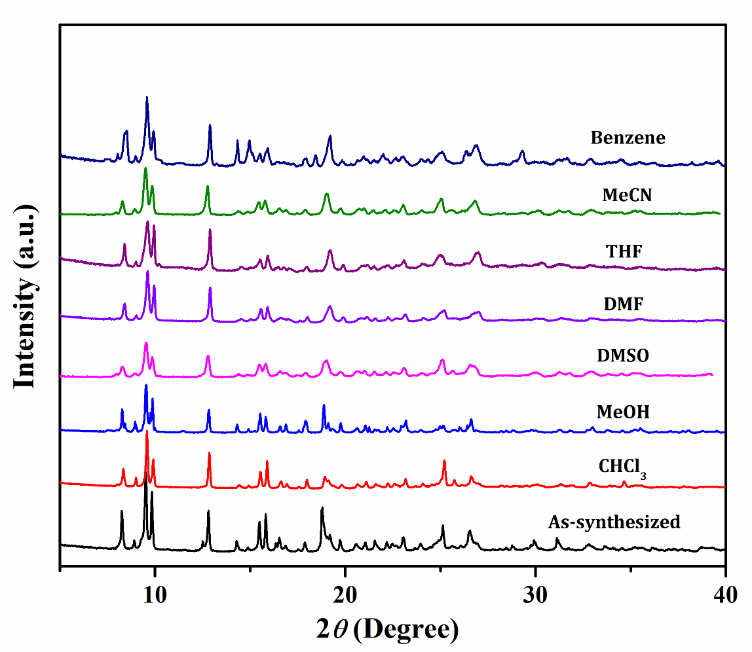


**Figure S5**: The PXRD pattern of **IITKGP-71** after immersing in different organic solvents shows an indistinguishable pattern from that of the as-synthesized pattern.


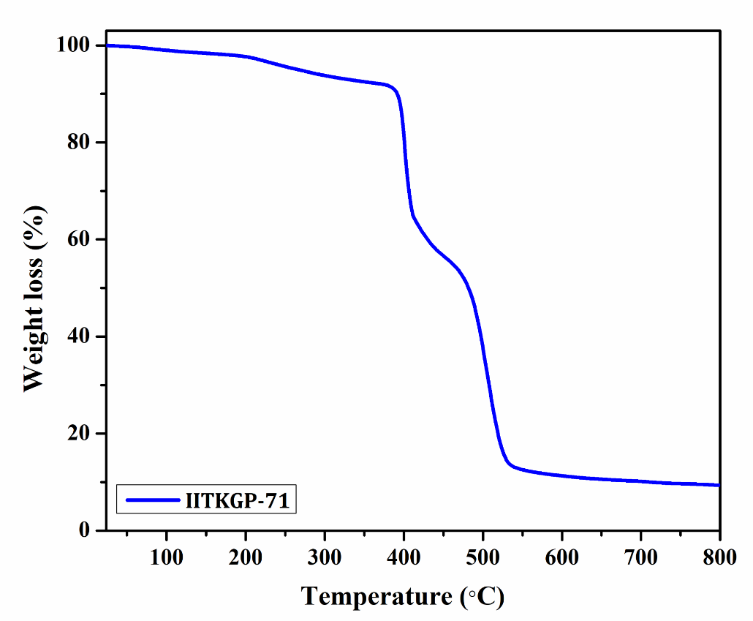


**Figure S6**: TGA plot of as-synthesized **IITKGP-71** within the temperature range of 25-800 °C under N_2_-atmosphere, showing thermal stability up to 375 °C.


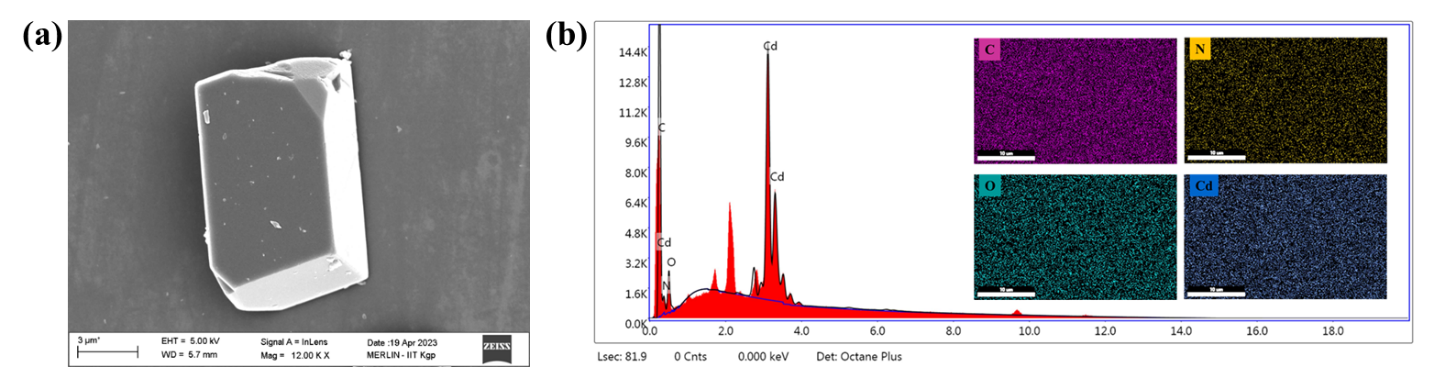


**Figure S7**: (a) FESEM images of as-synthesized **IITKGP-71**; (b) EDX and elemental mapping of **IITKGP-71** displaying the homogeneous distribution of the constituent elements on MOF surface.


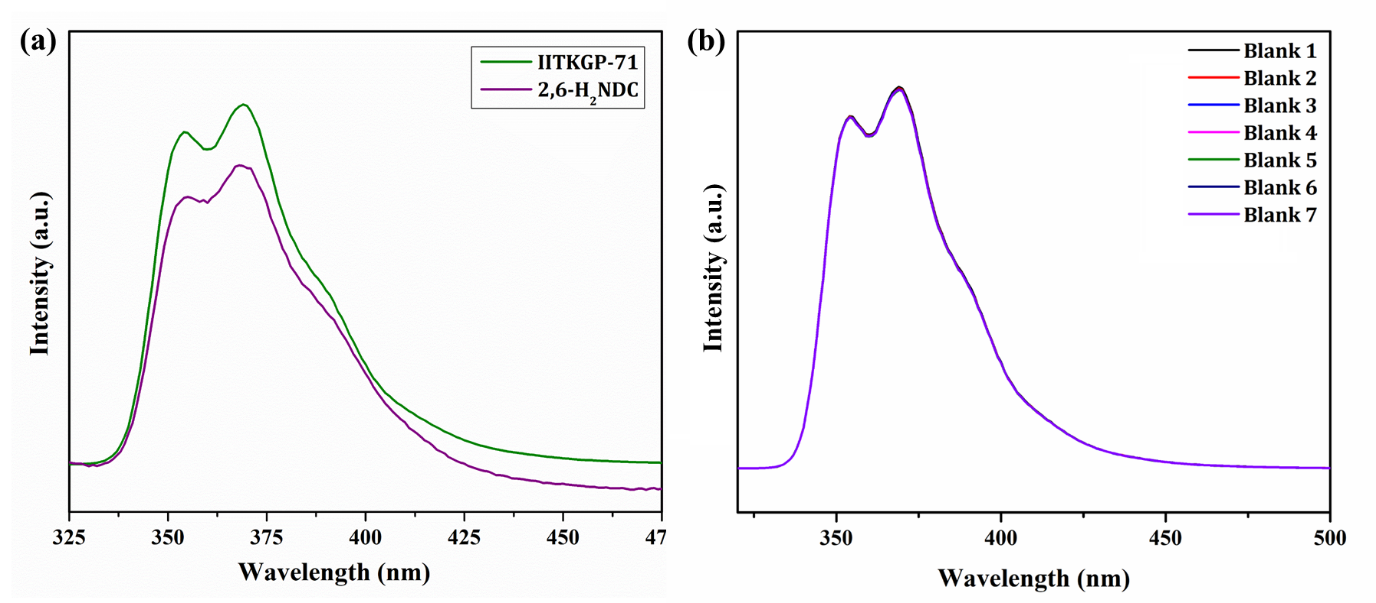


**Figure S8**: (a) Spectral overlap between the emission spectrum of **IITKGP-71** and 2,6-H_2_NDC (suspension in water) upon excitation at 280 and 284 nm, respectively; (b) luminescence spectra of blank solution of **IITKGP-71** with multiple measurements.


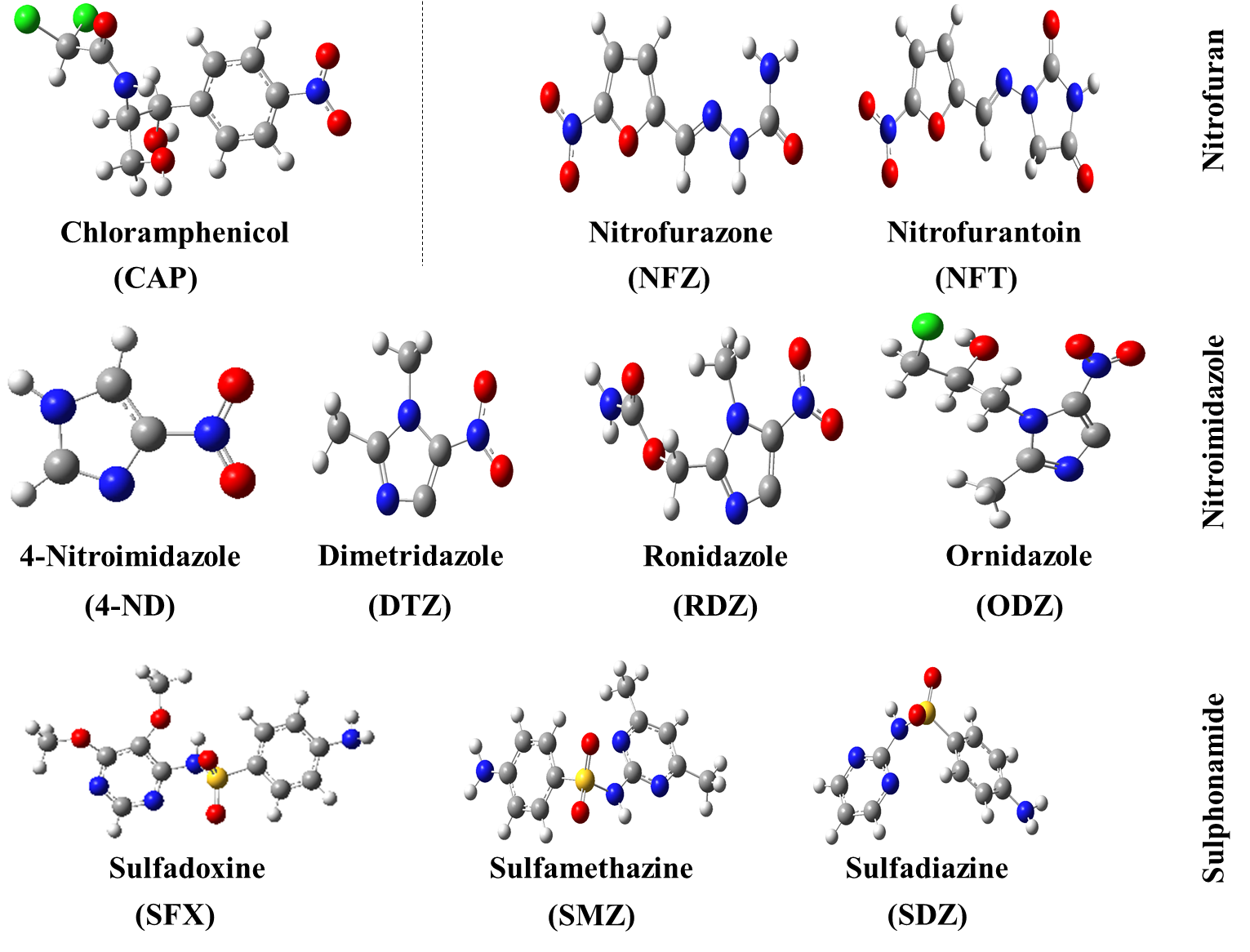


**Figure S9**: Optimized structures of the antibiotics.


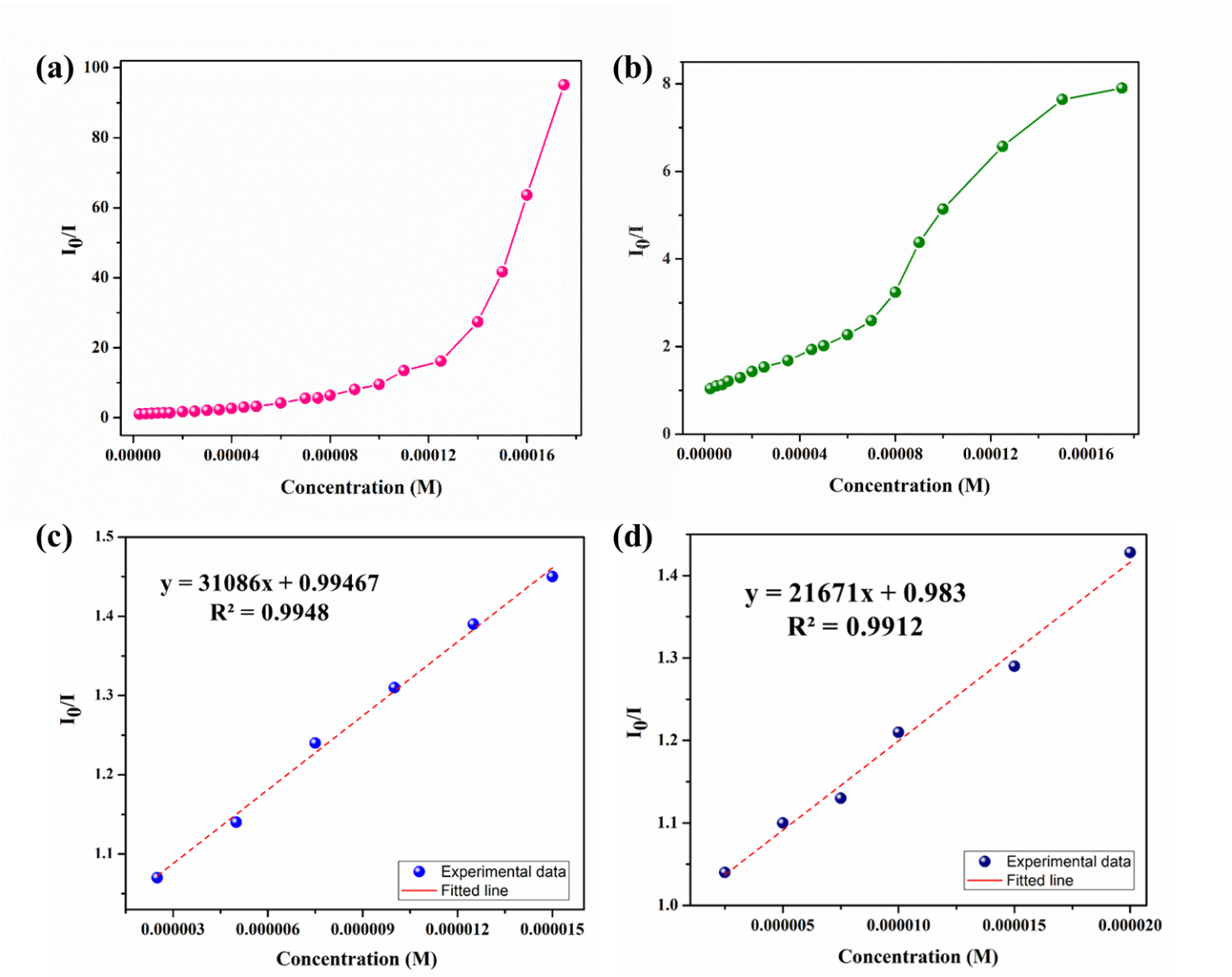


**Figure S10**: Stern−Volmer plot for **IITKGP-71** with a gradual increase in the concentration of (a) NFZ and (b) NFT antibiotics in water; linear region of luminescence intensity of probe in the low concentration range upon addition of (c) NFZ and (d) NFT analytes, respectively.


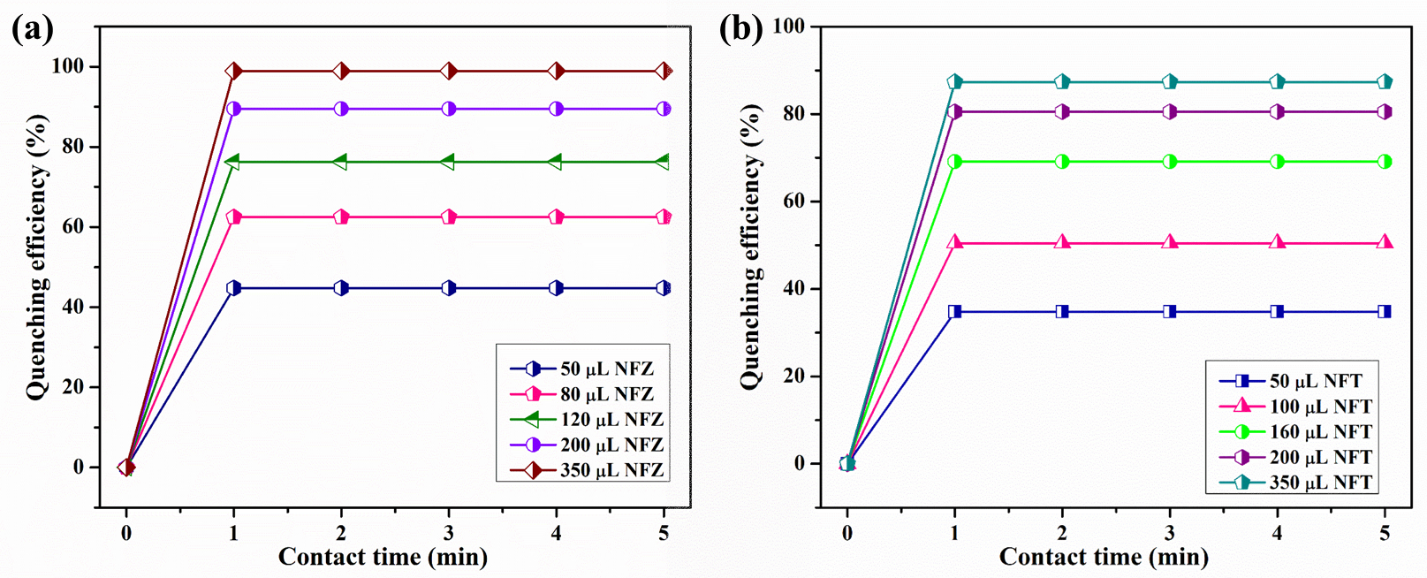


**Figure S11**: Kinetic plots for (a) NFZ and (b) NFT antibiotics sensing, respectively.


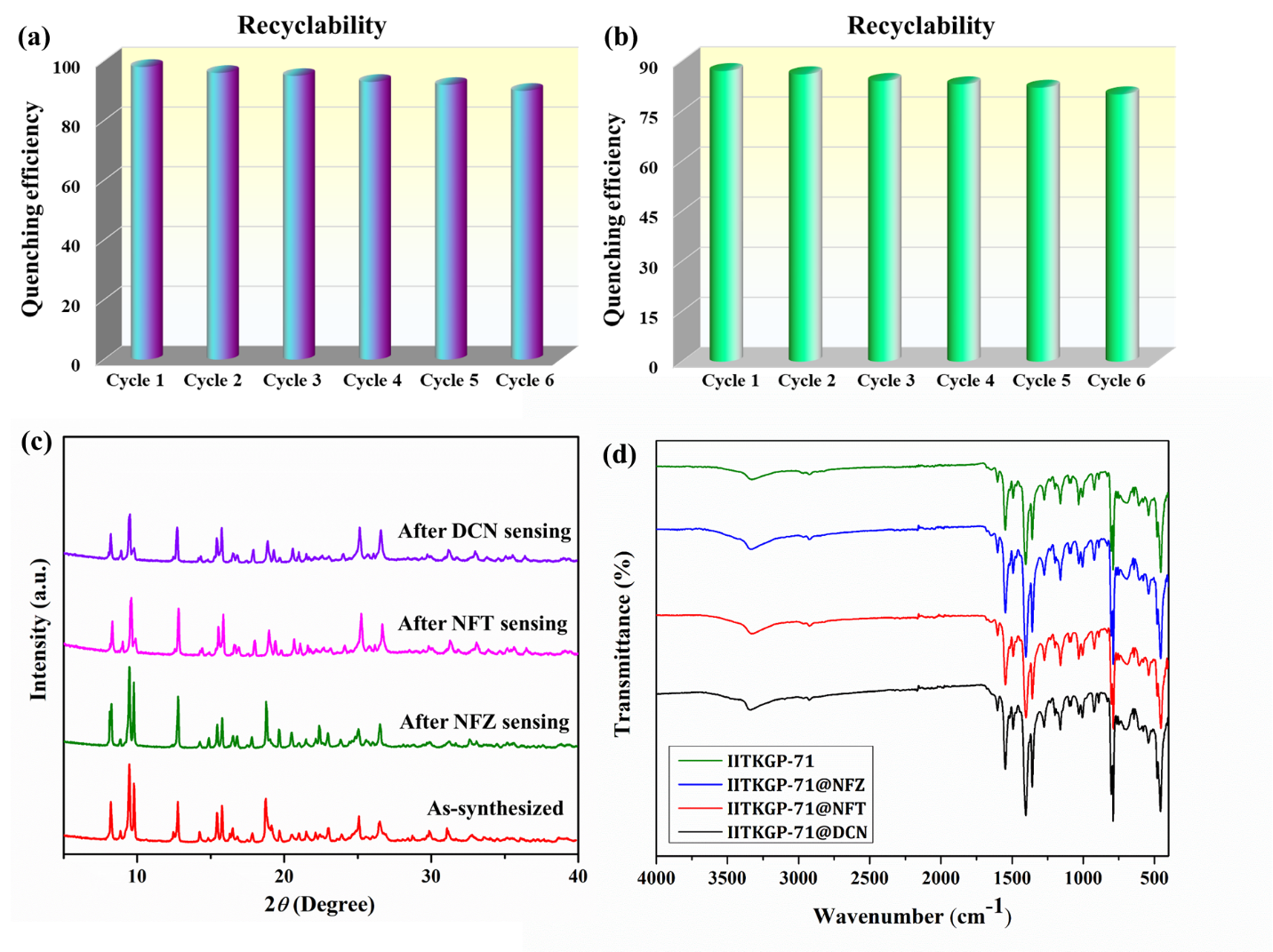


**Figure S12**: Recyclability plots after (a) NFZ and (b) NFT antibiotics sensing, respectively; Comparison of (c) PXRD patterns and (d) IR spectra of **IITKGP-71** after NFZ and NFT antibiotics and DCN pesticide sensing, with that of the as-synthesized material.


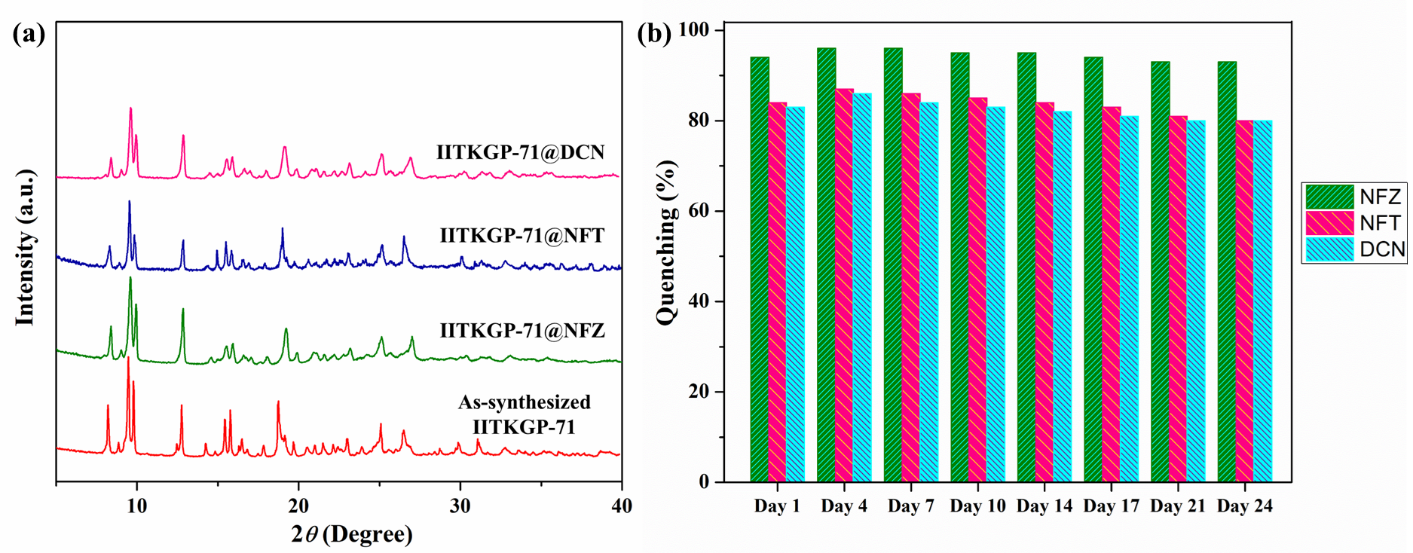


**Figure S13**: (a) Long-term stability (after two weeks of immersion) in NFZ, NFT and DCN analyte solutions; (b) long-term luminescence quenching study of NFZ, NFT antibiotics and DCN pesticide analytes.

**
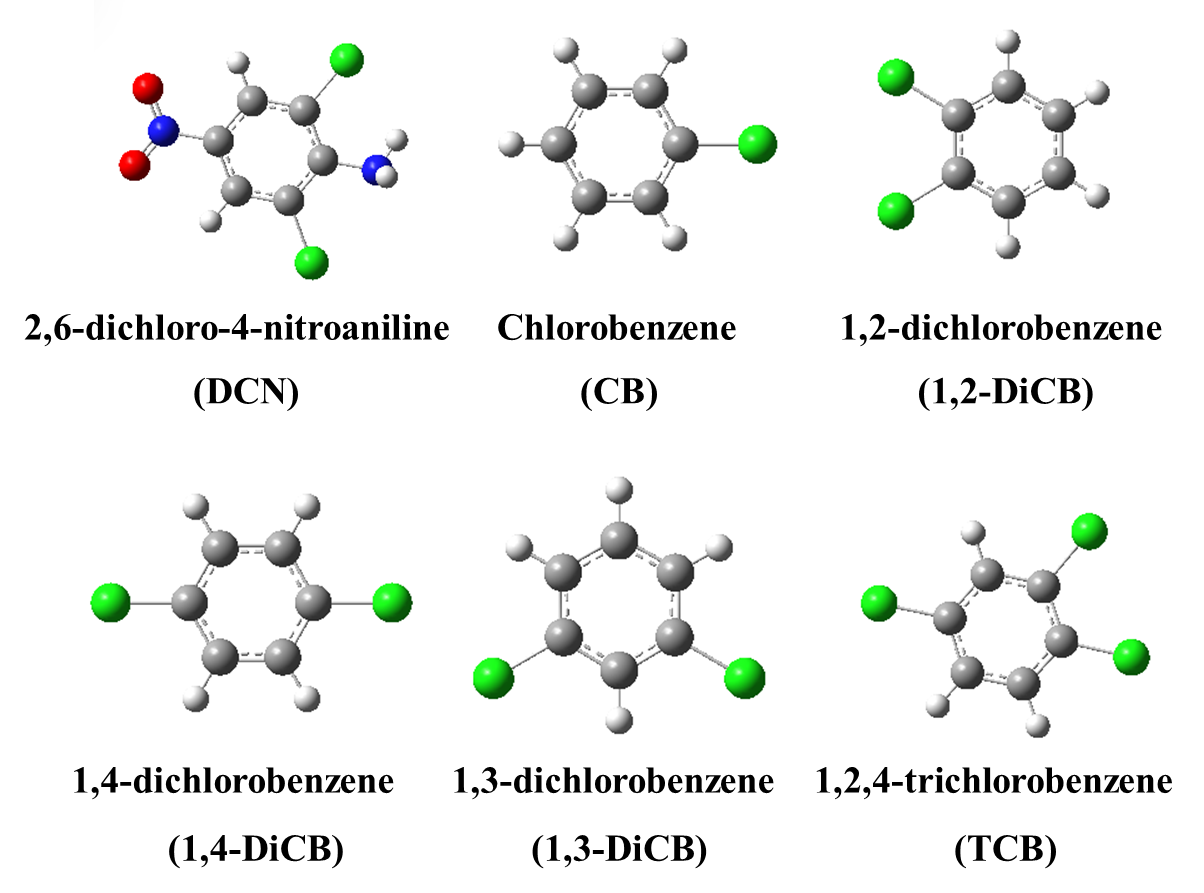
**

**Figure S14**: Optimized structures of the pesticides.


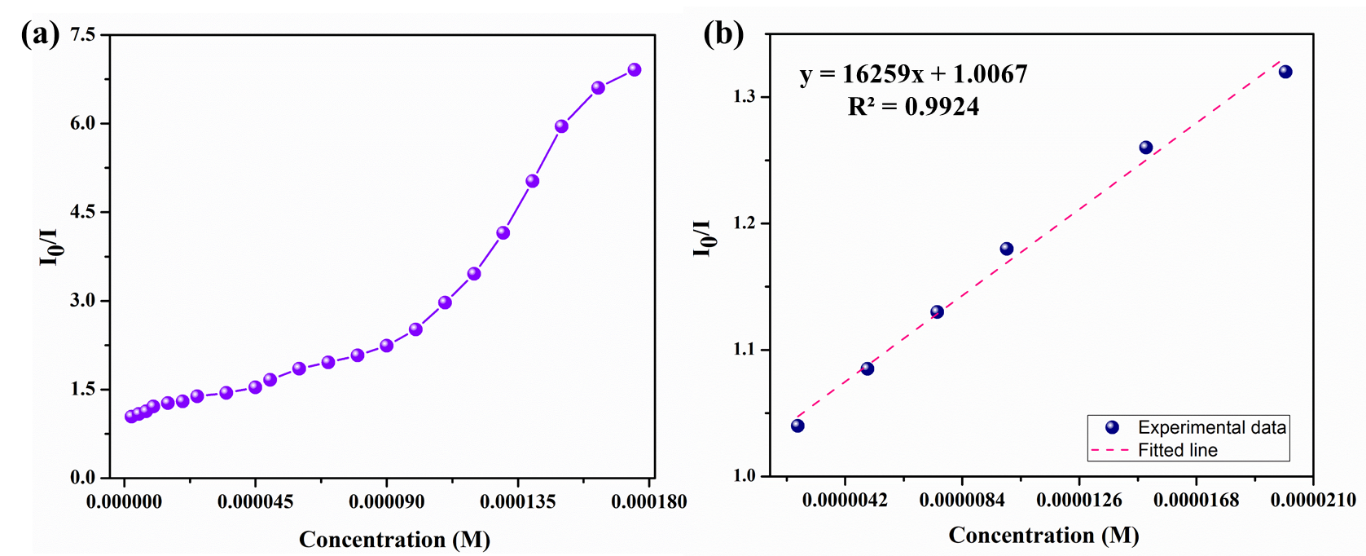


**Figure S15**: (a) Stern−Volmer plot for **IITKGP-71** with a gradual increase in the concentration of DCN; (b) linear region of luminescence intensity of probe in the low concentration range upon addition of DCN.


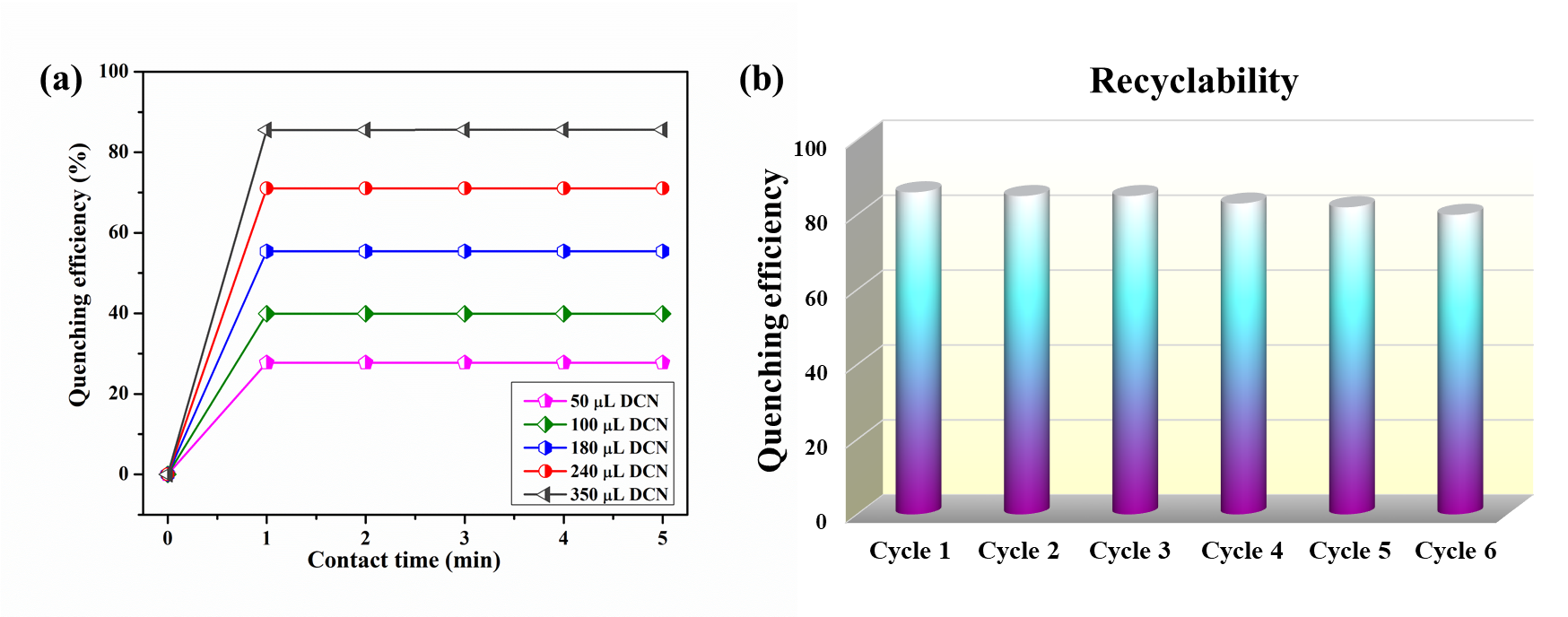


**Figure S16**: (a) Kinetic and (b) recyclability plots for DCN sensing.

**
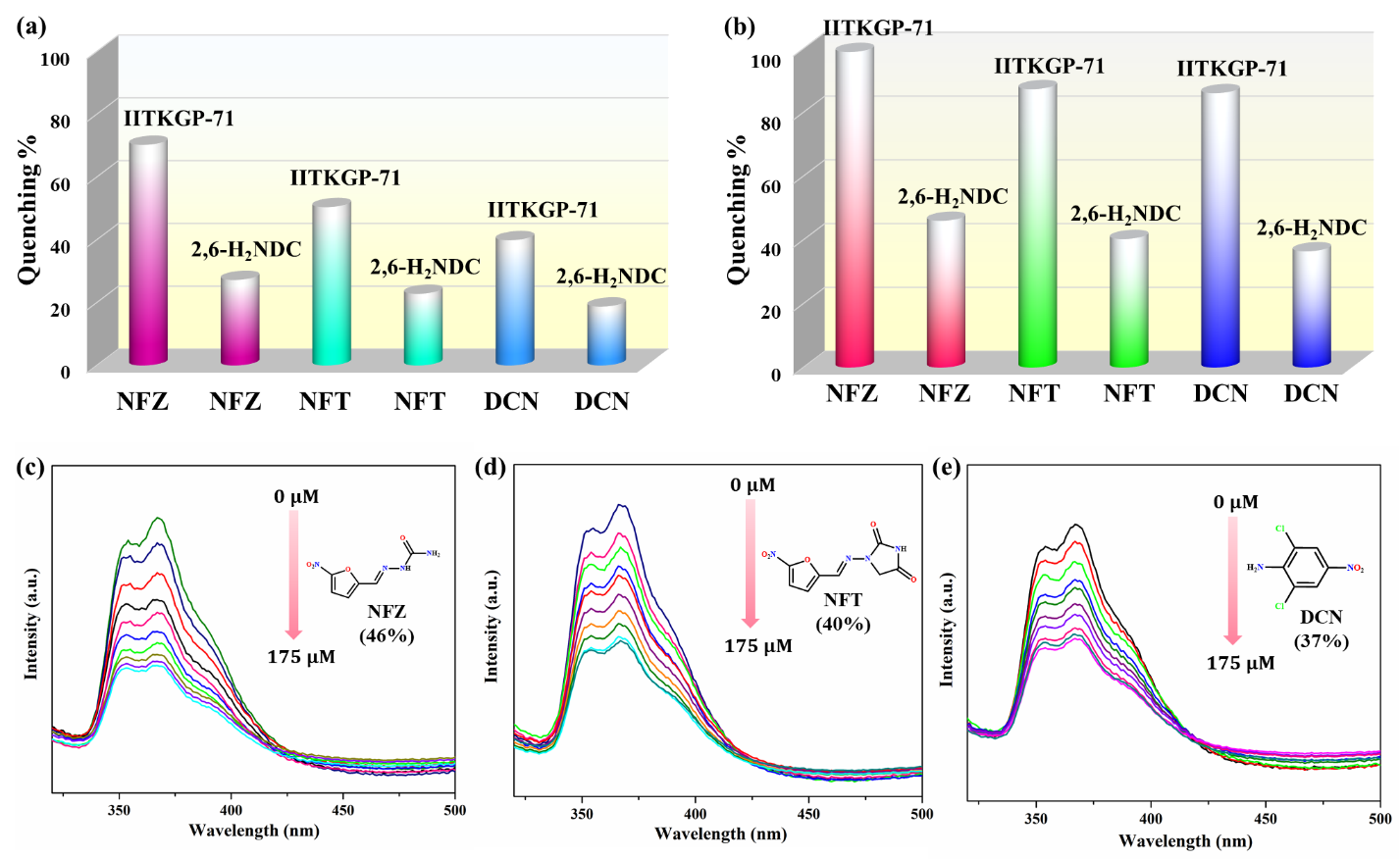
**

**Figure S17**: Comparison of detection ability of pristine MOF **IITKGP-71** with that of 2,6-H_2_NDC linker upon addition of (a) 50 µM and (b) 175 µM NFZ, NFT and DCN analytes; Change in the luminescence intensity of only ligand 2,6-H_2_NDC after gradual addition of (c) NFZ, (d) NFT and (e) DCN analytes, respectively.


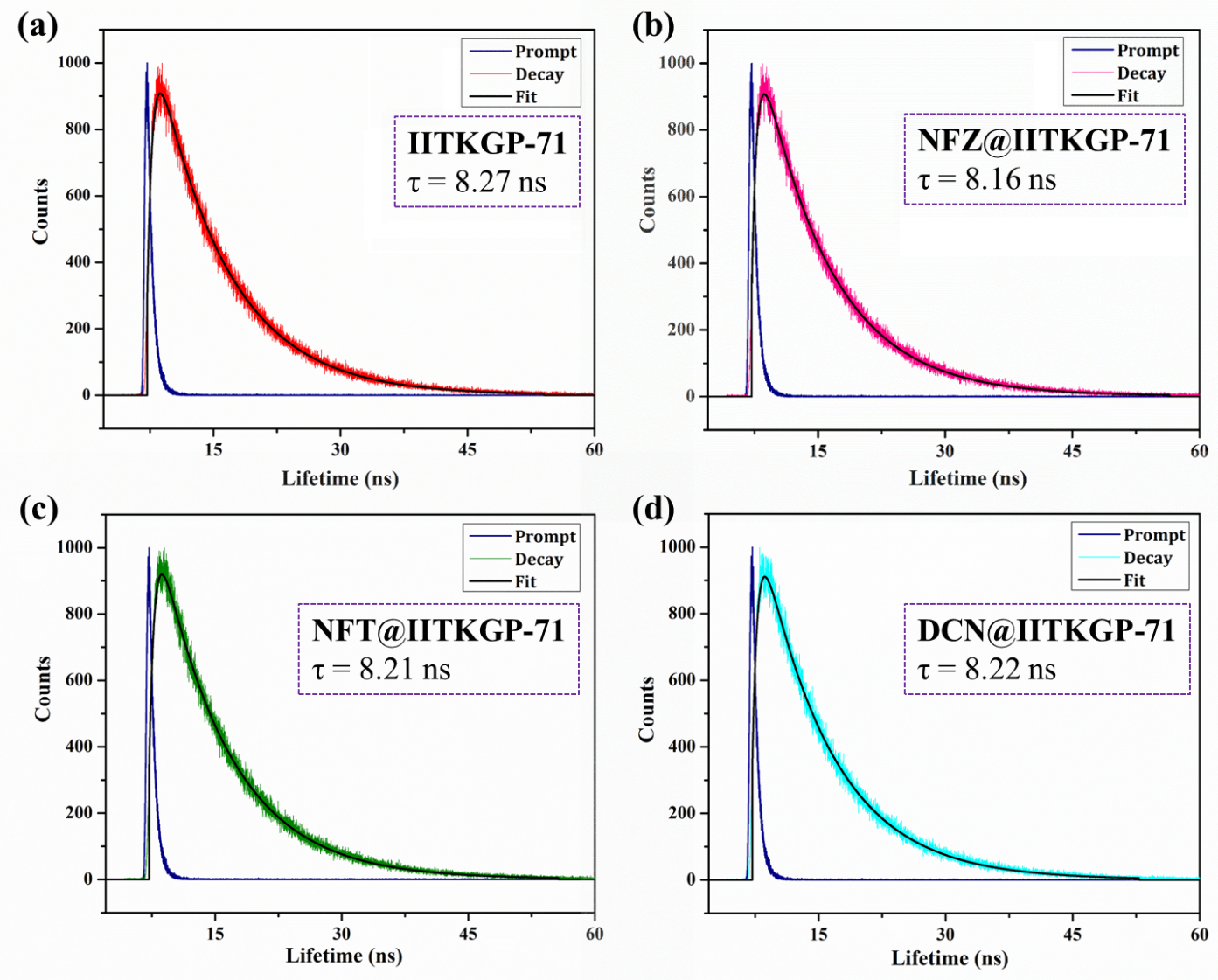


**Figure S18**: Lifetime decay profile of **IITKGP-71** (a) before and after (b) NFZ, (c) NFT, and (d) DCN addition, respectively.


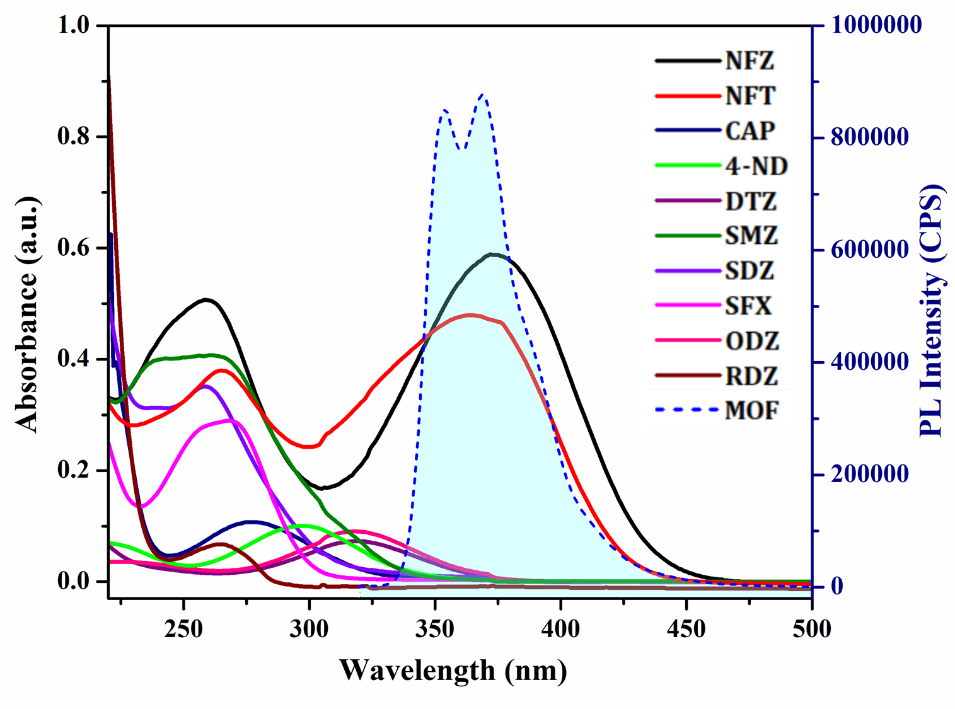


**Figure S19**: Spectral overlaps between the UV-vis absorption spectrum of NFZ, NFT, and other antibiotic analytes with the emission spectra of **IITKGP-71** in water.


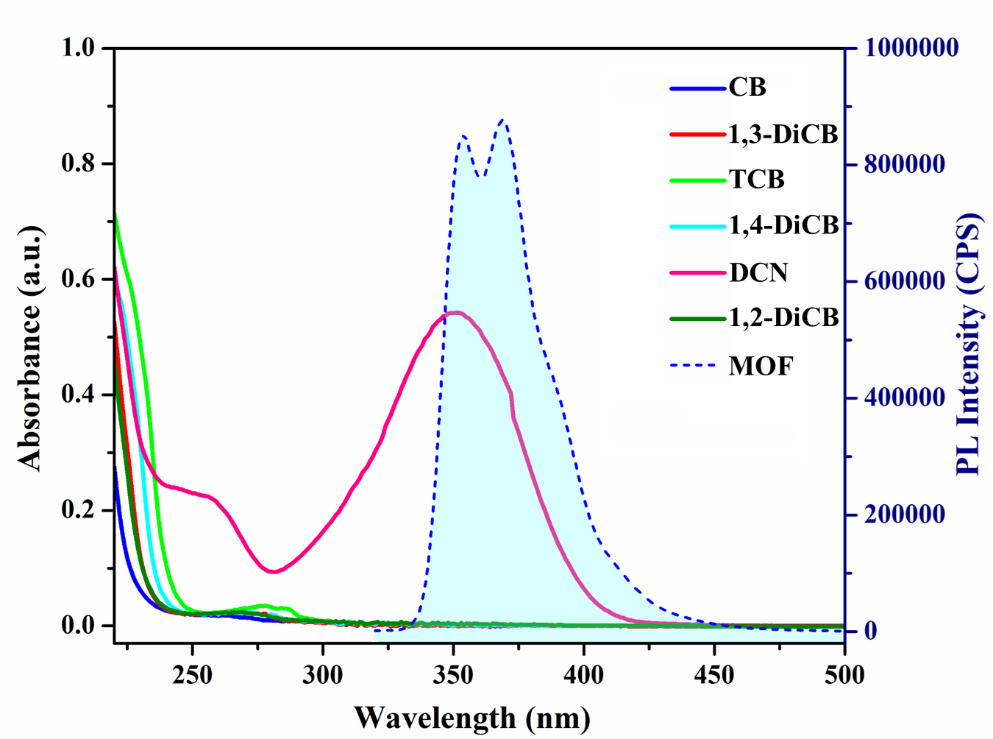


**Figure S20**: Spectral overlaps between the UV-vis absorption spectrum of DCN and other pesticide analytes with the emission spectra of **IITKGP-71** in water.


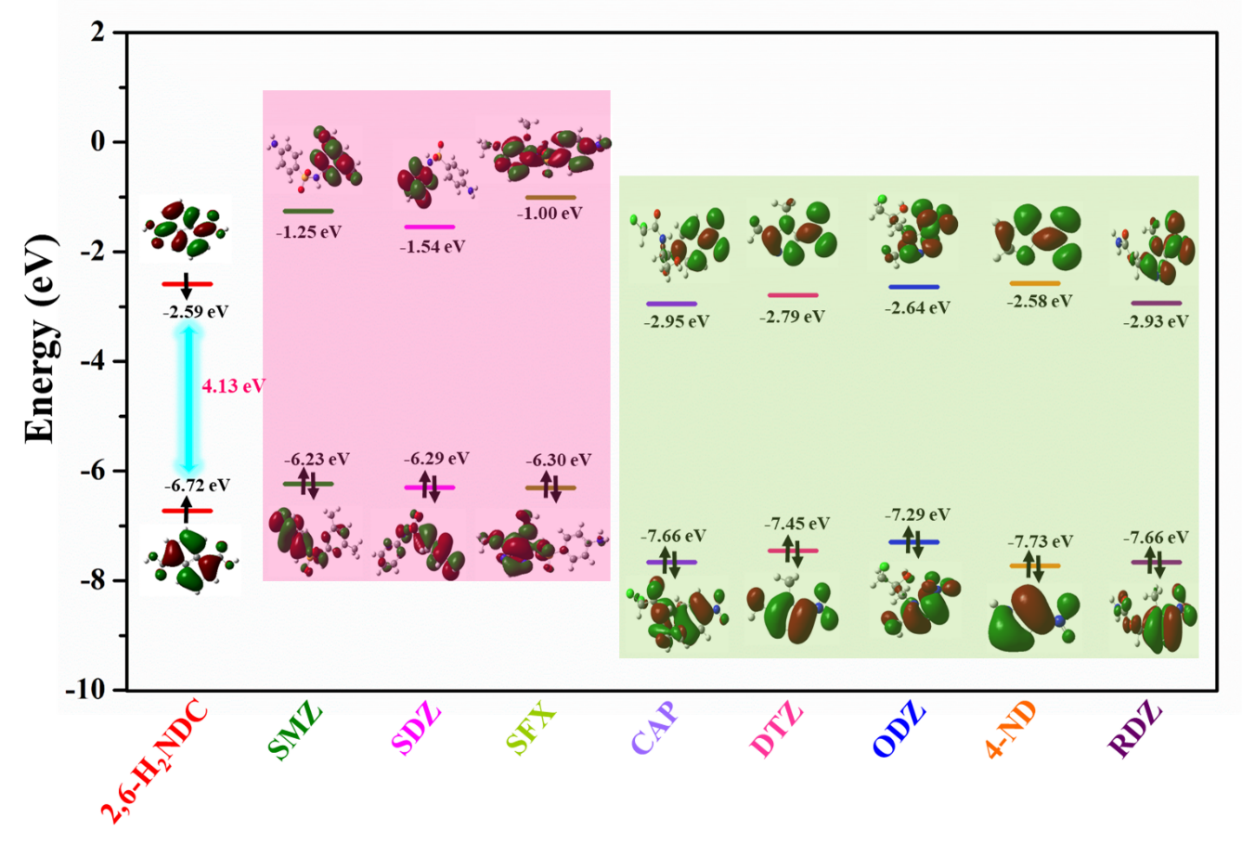


**Figure S21**: Comparison of HOMO-LUMO orbitals of other antibiotics with that of the 2,6-H_2_NDC acid linker.


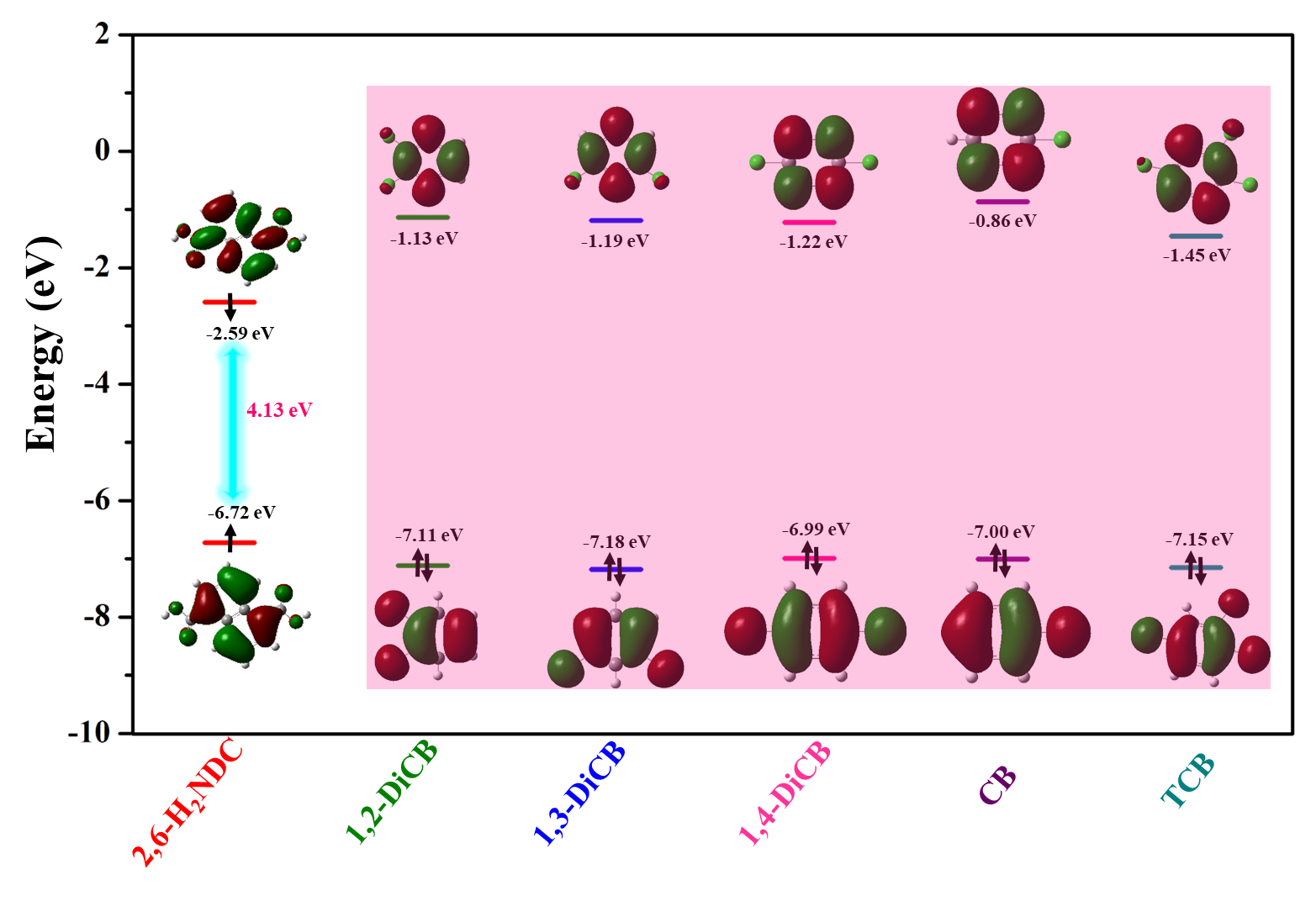


**Figure S22**: Comparison of HOMO-LUMO orbitals of the interfering pesticides with that of the 2,6-H_2_NDC acid linker.

**Table S1:** Crystal data and structure refinements for as-synthesized **IITKGP-71** and water treated MOF**, IITKGP-71w**.

|  | **IITKGP-71** | **IITKGP-71w** |
| --- | --- | --- |
| Empirical formula | C_23_ H_26_ Cd N_4_ O_6_ | C_23_ H_26_ Cd N_4_ O_6_ |
| Formula weight | 566.88 | 566.88 |
| Temperature (K) | 296 | 120 |
| Radiation | *Mo (k_α_)* | *Mo (k_α_)* |
| Wavelength (Å) | 0.71073 | 0.71073 |
| Crystal system | Triclinic | Triclinic |
| Space group | *P*$\bar{1}$ | *P*$\bar{1}$ |
| *a* [Å] | 10.940(5) | 10.952(5) |
| *b* [ Å] | 11.594(5) | 11.516(5) |
| *c* [ Å] | 11.999(4) | 11.940(6) |
| *α* [°] | 104.52(3) | 104.921(17) |
| *β* [°] | 104.73(3) | 104.897(10) |
| *γ* [°] | 112.21(4) | 111.749(10) |
| Volume [Å^3^] | 1256.3(10) | 1249.9(10) |
| Z | 2 | 2 |
| Density (calculated) [g cm^-3^] | 1.499 | 1.515 |
| Absorption coefficient [mm^-1^] | 0.913 | 0.923 |
| *F (000)* | 576 | 576 |
| Refl. used [*I* > 2*σ*(*I*)] | 5784 | 4290 |
| Independent reflections | 11092 | 4977 |
| *R*_int_ | 0.0575 | 0.1026 |
| Refinement method | full-matrix least squares on *F*^2^ | full-matrix least squares on *F*^2^ |
| GOF | 1.037 | 1.076 |
| Final *R* indices[*I*>2σ(I)] | *R_1_*=0.0661 *wR_2_*=0.1528 | *R_1_*=0.0563 *wR_2_*=0.1483 |
| *R* indices (all data) | *R_1_*=0.1486 *wR_2_*=0.2024 | *R_1_*=0.0679 *wR_2_*=0.1564 |
| *CCDC* | 2388546 | 2401218 |

**Table S2:** Selected Bond Distances (Å) and Bond Angles (^o^) in **IITKGP-71**.

Bond Distances (Å)

| Cd1-O1 | 2.335(5) | Cd1-O3 | 2.491(5) |
| --- | --- | --- | --- |
| Cd1-O2 | 2.356(5) | Cd1-O4 | 2.322(5) |
| Cd1-N1 | 2.247(5) | Cd1-N4#1 | 2.258(5) |
| OW1-H1W1 | 0.85 | OW2-H1W2 | 0.85 |
| OW1-H2W1 | 0.85 | OW2-H2W2 | 0.85 |
| #1: -x,1-y,1-z | | | |

Bond Angles (^o^)

| O1-Cd1-O2 | 55.45(16) | O1- Cd1-O3 | 148.48(18) |
| --- | --- | --- | --- |
| O1-Cd1-O4 | 94.66(18) | O3- Cd1-O4 | 53.95(16) |
| O1-Cd1-N1 | 126.94(19) | O3- Cd1-N1 | 83.88(16) |
| O1-Cd1-N4#1 | 84.78(17) | O3- Cd1-N4#1 | 103.38(17) |
| O2-Cd1-O3 | 114.86(16) | O4- Cd1-N1 | 133.34(16) |
| O2- Cd1-O4 | 89.59(17) | O4- Cd1-N4#1 | 102.83(17) |
| O2- Cd1-N1 | 97.30(17) | N1- Cd1-N4#1 | 101.27(17) |
| O2- Cd1-N4#1 | 139.38(16) |  |  |
| #1: -x,1-y,1-z | | | |

**Table S3:** Hydrogen-bonding interactions in **IITKGP-71**.

| **D ̶ H···A** | **d(H···A) (Å)** | **d(D···A) (Å)** | **∠DHA (°)** |
| --- | --- | --- | --- |
| N3- H3···O4 | 2.257 | 3.052 | 153.56 |
| OW2- H1W2···OW1 | 2.32 | 3.058 | 145.51 |
| OW1- H2W1···OW2 | 2.41 | 3.058 | 133.48 |
| N2- H2···O3 | 2.231 | 2.847 | 128.47 |
| N3- H3···O1 | 2.521 | 3.048 | 120.30 |

**Table S4:** Non-bonding interactions in **IITKGP-71**.

| **D ̶ H···A** | **d(H···A) (Å)** | **d(D···A) (Å)** | **∠DHA (°)** |
| --- | --- | --- | --- |
| C21- H21C···O3 | 3.116 | 3.864 | 135.96 |
| C23- H23B···O2 | 2.701 | 3.608 | 158.2 |
| C3- H3A···O4 | 2.824 | 3.708 | 159.28 |
| C3- H3A···O3 | 3.429 | 4.149 | 136.01 |
| C16- H16C···O4 | 3.004 | 3.927 | 161.7 |
| C16- H16C···O3 | 2.932 | 3.791 | 149.7 |
| C6- H6···OW1 | 3.338 | 3.836 | 143.19 |
| C10- H10A···OW1 | 3.051 | 3.886 | 119.89 |

**Table S5:** Comparison table of quenching constants, their Limit of Detection (LOD) of previously reported CP/MOFs for sensing of NFT and NFZ in aqueous medium.

| **Sl. No** | **MOF/CP** | **Analyte** | **Quenching efficiency constant (M^-1^)** | **LOD (μM)** | **Ref.** |
| --- | --- | --- | --- | --- | --- |
| 1. | **IITKGP-71** | **NFT**  **NFZ** | **2.1×10^4^**  **3.1×10^4^** | **0.17**  **0.11** | **This work** |
| 2. | [Eu(H_2_DHTA)_0.5_(DHTA)_0.5_(DMF)(H_2_O)]·2H_2_O | NFT  NFZ | 7.51×10^4^  5.95×10^4^ | 0.22  0.26 | *Inorg. Chem.* **2024**, *63*, 18058. |
| 3. | {[(CH_3_)_2_NH_2_]_2_[Pb(TCBPE)(H_2_O)_2_]}*_n_* | NFT  NFZ | 2.01×10^4^  1.934×10^4^ | 0.33  0.35 | *ACS Appl. Mater. Interfaces* **2022***, 14,* 51531. |
| 4. | Cd-CP | NFT  NFZ | 1.33×10^4^  3.57×10^4^ | 4.06 nM  1.51 nM | *Journal of Molecular Structure* **2024**, *1315*, 138875. |
| 5. | Eu-CMOF | NFT  NFZ | 2.24×10^3^  6.41×10^3^ | 2.80×10^-6^ M  1.33×10^-6^ M | *Cryst. Growth Des.* **2022**, *22*, 3991. |
| 6. | [Zn_2_(L)_2_(4,4’-bpy)]∙4DMF | NFT  NFZ | 1.14×10^4^  1.30×10^4^ | 2.0  2.5 | *J. Solid State Chem.* **2020***, 286,* 121318. |
| 7. | [Zn(L)(phen)]·0.5DMF | NFT  NFZ | 1.27×10^4^  1.45×10^4^ | 6.7  5.4 |  |
| 8. | [Cd_2_Cl(L)(H_2_O)]·11H_2_O | NFT  NFZ | 1.5×10^4^  2.1×10^4^ | 0.26  0.2 | *Dalton Trans.* **2020***, 49,* 7488. |
| 9. | RhB@Tb-dcpcpt | NFT  NFZ | 6.69×10^4^  5.98×10^4^ | 0.448  0.502 | *ACS Appl. Mater. Interfaces* **2019**, *11*, 21201. |
| 10. | FCS-4 | NFT  NFZ | 1.32×10^4^  1.13×10^4^ | 0.53 ppm  0.73 ppm | *Inorg. Chem. Front.* **2021**, *8*, 1290. |
| 11. | FCS-5 | NFT  NFZ | 5.43×10^4^  3.74×10^4^ | 0.15 ppm  0.22 ppm |  |
| 12. | Eu-BCA | NFT  NFZ | 1.6×10^4^  2.2×10^4^ | 0.21  0.16 | *Chem. Commun.* **2015***, 51,* 8300. |
| 13. | BUT-12 | NFT  NFZ | 3.8×10^4^  3.1×10^5^ | --  0.17 | *J. Am. Chem. Soc.* **2016***, 138,* 6204. |
| 14. | BUT-13 | NFT  NFZ | 6.0×10^4^  7.5×10^5^ | --  0.46 | *J. Am. Chem. Soc.* **2016***, 138,* 6204. |
| 15. | RhB-CDs@1 | NFT  NFZ | 1.98×10^4^  2.09×10^4^ | 0.33  0.31 | *J. Mater. Chem. C* **2019***, 7,* 15057. |

**Table S6:** Comparison table of quenching constants, their Limit of Detection (LOD) of previously reported CP/MOFs for sensing of DCN in different solvent medium.

| **Sl. No** | **MOF/CP** | **Quenching efficiency Constant (M^-1^)** | **LOD (μM)** | **Medium** | **Ref.** |
| --- | --- | --- | --- | --- | --- |
| 1. | **IITKGP-71** | **1.6× 10^4^** | **0.21** | **Water** | **This work** |
| 2. | CdMOF-1 | 4.93×10^4^ | 0.36 | Water | *ACS Appl. Mater. Interfaces* **2023**, *15*, 6177. |
| 3. | CdMOF-2 | 2.03×10^4^ | 0.12 | Water |  |
| 4. | CSMCRI-9 | 4.96×10^4^ | 95 nM | Water | *Mater. Chem. Front.* **2021**, *5*, 979. |
| 5. | Mg-APDA | 7.5×10^4^ | 150  ppb | DMF | *Inorg. Chem.* **2018***, 57,* 13330. |
| 6. | [Zn_3_(DDB)(DPE)]·H_2_O | 3.3×10^4^ | 2.7×10^-7^ M | Water | *Dalton Trans.* **2019***, 48,* 16776. |
| 7. | [Eu_2_(dtztp)(OH)_2_(DMF)(H_2_O)_2.5_]·2H_2_O | 6.25×10^4^ | 5.28  ppm | Water | *Sensors and Actuators B: Chemical* **2021***, 331,* 129377. |
| 8. | [Ag(CIP^−^)] | 5.2×10^4^ | 1.7×10^-7^ M | DMF | *Dalton Trans.* **2019***, 48,* 10892*.* |
| 9. | JXUST-12 | 4.75×10^4^ | 0.88 | EtOH | *Inorg. Chem. Front.* **2022***, 9,* 1504. |
| 10. | JXUST-12a | 4.75×10^4^ | 0.34 | EtOH |  |
| 11. | [Zn_2_(bpdc)_2_(BPyTPE)] | -- | 0.63 | DCM | *Chem. Commun.* **2017***, 53*, 9975. |
| 12. | F3Y | 3.73×10^4^ | 0.14 | Water | *ACS Appl. Mater. Interfaces* **2022***, 14,* 51531. |
| 13. | [Cd_3_(CBCD)_2_(DMA)_4_(H_2_O)_2_]·10DMA | 4.47×10^4^ | 145 ppb | DMA | *Dalton Trans.* **2019***, 48,* 2683. |
| 14. | {Zn_4_(TPOM)(1,4-NDC)_4_}*_n_* | 2.74 × 10^4^ | 0.28 ppm | Water | *ACS Appl. Mater. Interfaces* **2018**, *10*, 42406. |
| 15. | (H_3_O)[Zn_2_L(H_2_O)]·3NMP·6H_2_O | 1.12 × 10^5^ | 2.93 ppm | NMP | *RSC Adv.* **2019**, *9*, 38469. |
| 16. | Eu^3+^@Zn-MOF-B | 3.3 × 10^4^ | 0.89 μM | Water | *Inorg. Chem.* **2022**, *61*, 23, 8966. |
| 17. | Eu^3+^@Zn-MOF-NS | 3.2 × 105 | 0.17 μM | Water | *Inorg. Chem.* **2022**, *61*, 23, 8966. |
| 18. | [Zn_2_(L)_2_(TPA)].2H_2_O | 2.36 × 10^4^ | 0.39 ppm | Methanol | *New J. Chem.* **2019**, *43*, 2353. |

**References:**

1. Sheldrick, G. M. Siemens Area Correction Absorption Correction Program; University of Göttingen: Göttingen, Germany, **1994**.
2. Farrugia, L. J. WinGx suite for small-molecule single crystal crystallography. *J. Appl. Crystallogr.* **1999**, *32*, 837.
3. SAINT+, 6.02ed, Bruker AXS, Madison, WI, **1999**,
4. XPREP, 5.1 ed. Siemens Industrial Automation Inc., Madison, WI, **1995**,
5. Sheldrick, G. M. SHELXL-97 Program for Crystal Structure Solution and Refinement; University of Göttingen: Göttingen, Germany, **1997**.
6. Sheldrick, G. M. Crystal Structure Refinement with SHELXL. *Acta Cryst C* **2015**, *71*, 3.
7. Mondal, S.; Pramanik, B.; Sahoo, R.; Das M. C. *ChemSusChem* **2024**, e202401248.
